# Guts within guts: the microbiome of the intestinal helminth parasite *Ascaris suum* is derived but distinct from its host

**DOI:** 10.1186/s40168-022-01399-5

**Published:** 2022-12-16

**Authors:** Ankur Midha, Víctor Hugo Jarquín-Díaz, Friederike Ebner, Ulrike Löber, Rima Hayani, Arkadi Kundik, Alessio Cardilli, Emanuel Heitlinger, Sofia Kirke Forslund, Susanne Hartmann

**Affiliations:** 1 Department of Veterinary Medicine, Center for Infection Medicine, Institute of Immunology, Freie Universität Berlin, Robert-von-Ostertag-Straße 7, 14163 Berlin, Germany; 2 Experimental and Clinical Research Center, a cooperation between the Max-Delbrück-Center for Molecular Medicine in the Helmholtz Association and the Charité — Universitätsmedizin Berlin, Berlin, Germany; 3 Charité — Universitätsmedizin Berlin, corporate member of Freie Universität Berlin and Humboldt-Universität zu Berlin, Lindenberger Weg 80, 13125 Berlin, Germany; 4 Max-Delbrück-Center for Molecular Medicine in the Helmholtz Association (MDC), Berlin, Germany; 5 Department of Molecular Parasitology, Institute for Biology, Humboldt-Universität zu Berlin, Philippstraße 13, 10115 Berlin, Germany; 6 Research Group Ecology and Evolution of Molecular Parasite-Host Interactions, Leibniz-Institute for Zoo and Wildlife Research (IZW), Alfred-Kowalke-Straße 17, 10315 Berlin, Germany; 7 Structural and Computational Biology Unit, European Molecular Biology Laboratory, Meyerhofstraße 1, 69117 Heidelberg, Germany; 8 Berlin Institute of Health, Berlin, Germany; 9 DZHK (German Centre for Cardiovascular Research), Partner Site Berlin, Berlin, Germany

## Abstract

**Background:**

Intestinal helminths are extremely prevalent among humans and animals. In particular, intestinal roundworms affect more than 1 billion people around the globe and are a major issue in animal husbandry. These pathogens live in intimate contact with the host gut microbiota and harbor bacteria within their own intestines. Knowledge of the bacterial host microbiome at the site of infection is limited, and data on the parasite microbiome is, to the best of our knowledge, non-existent.

**Results:**

The intestinal microbiome of the natural parasite and zoonotic macropathogen, *Ascaris suum* was analyzed in contrast to the diversity and composition of the infected host gut. 16S sequencing of the parasite intestine and host intestinal compartments showed that the parasite gut has a significantly less diverse microbiome than its host, and the host gut exhibits a reduced microbiome diversity at the site of parasite infection in the jejunum. While the host’s microbiome composition at the site of infection significantly determines the microbiome composition of its parasite, microbial signatures differentiate the nematodes from their hosts as the *Ascaris* intestine supports the growth of microbes that are otherwise under-represented in the host gut.

**Conclusion:**

Our data clearly indicate that a nematode infection reduces the microbiome diversity of the host gut, and that the nematode gut represents a selective bacterial niche harboring bacteria that are derived but distinct from the host gut.

Video Abstract

**Supplementary Information:**

The online version contains supplementary material available at 10.1186/s40168-022-01399-5.

## Introduction

The gastrointestinal ecosystem contains a diverse community of viral, prokaryotic (bacteria & archaea), and eukaryotic (helminths & protozoa) components, the latter being recognized mainly as parasites. Understanding host-parasite interactions in this complex environment requires knowledge on the dynamics between these community members. Bacteria and parasites share the same environment in the gut in which they alter host physiology and metabolism and at the same time provide crucial signals for the development and function of the host intestinal immune system [1–3]. Intestinal nematode infections are extremely widespread in humans as well as companion animals, livestock, and wildlife. Studies suggest that helminths may modify host-associated bacterial communities to modulate host immunity to promote their own successful establishment in the gut [4, 5]. Despite the close coexistence of helminths with numerous microbes, little is known concerning the reciprocal interactions of intestinal helminths with the microbiota and underlying mechanisms, and in particular, nothing is known about how parasite-associated microbiomes interact with the host microbiome and the host itself.

Several studies report alterations in the gut microbial composition of experimental and naturally helminth-infected murine, human, porcine, and other hosts [6, 7]; however, the consequences of these alterations are not elucidated. Dheilly et al. [8] proposed that parasites might benefit from modifications of host-associated microbiomes, which leads to immune modulation that reduces the resistance to infection. We showed earlier that infections with the murine nematode *Heligmosomoides polygyrus* alter the composition of the host-gut microbiota, and that the nematodes benefit from microbiota-induced immunomodulation [9, 10]. Others have also shown that alterations in bacterial composition during murine *H. polygyrus* and *Nippostrongylus brasiliensis* infection led to the induction of regulatory immune responses [11, 12], while increased short-chain fatty acid (SCFA) production was observed during numerous helminth infections, including in *Ascaris suum*-infected pigs [11]. Hence, microbes and their metabolites are involved in shaping the adaptive immune response directed against nematodes [10, 11]. Notably, intestinal nematodes also have a gut and are themselves colonized by bacteria [13, 14]; knowledge of the microbial inhabitants of intestinal helminths and their symbiotic and antagonistic relationships, however, remains elusive, despite recognition of parasite microbiomes as a key research target [15, 16]. There is an intimate trilateral interaction between intestinal nematodes, their microbial environment, and host cells [17], but almost nothing is known regarding parasite-associated microbiomes, and there is currently no human-relevant parasite microbiome available.

*Ascaris* is one of the most common and widespread intestinal parasites in humans and livestock. In tropical countries, the prevalence exceeds 10% of the population, causing around 60,000 deaths per year, malnutrition, and developmental deficits in children [18–21]. In pig husbandry, *Ascaris* leads to significant economic losses due to reduced feed conversion and liver condemnation at slaughter [22]. Although *Ascaris* exhibits a tissue migratory phase, it spends most of its lifetime in the gut sharing its environment with host-associated microbes that might present infectious threats or be beneficial by providing key nutrients, protecting against infections [23], promoting fecundity, or modulating host responses against *Ascaris* [17]. While there is some knowledge on alterations of the fecal microbiome of *Ascaris*-infected humans [24–26] as well as the porcine colonic and fecal microbiome during *Ascaris* infection [27, 28], the microbiome of *Ascaris* itself has not yet been studied. *Ascaris* produces various antimicrobial proteins and peptides which likely shape the microbiome in the immediate vicinity of, and within, the nematode itself [29]. Further insights into parasite-associated microbiomes could unveil novel strategies to control helminth infections [30]. Thus, we aimed to unravel the parasite microbiome and its interdependence with the host microbiome in which it exists. Our study indicates for the first time that a parasitic nematode’s microbiome is derived from microbes in its immediate vicinity but distinct in composition from the microbiome of the host.

## Methods

### Animals, infection trials, sampling, and DNA extraction

Intestinal content and worm samples were derived from two independent *A. suum* infection trials as well as noninfected animals. Infective *A. suum* eggs were collected and prepared as previously described [29]. In brief, slaughterhouse-derived adult female worms were cultured overnight, and released eggs were collected from culture fluid, washed, and incubated at room temperature in the dark for 6–8 weeks until > 90% embryonation rates were observed.

German Landrace pigs (*Sus scrofa*) from a conventional breeder aged 6 weeks were kept in separate groups and orally infected with 2000 (exp. 1) or 4000 (exp. 2) embryonated *A. suum* eggs/pig.

At 56 days post infection (DPI), pigs were sedated using ketamine hydrochloride and azaperone (20 mg/kg body weight [BW]; Ursotamin; Serumwerk Bernburg AG and 2 mg/kg BW; Stresnil; Janssen-Cilag GmbH, Germany) and euthanized by intracardial injection of T61 (10 mg/kg BW of tetracaine hydrochloride, mebezonium iodide, and embutramide, Intervet, Germany).

**Fig. 1:**
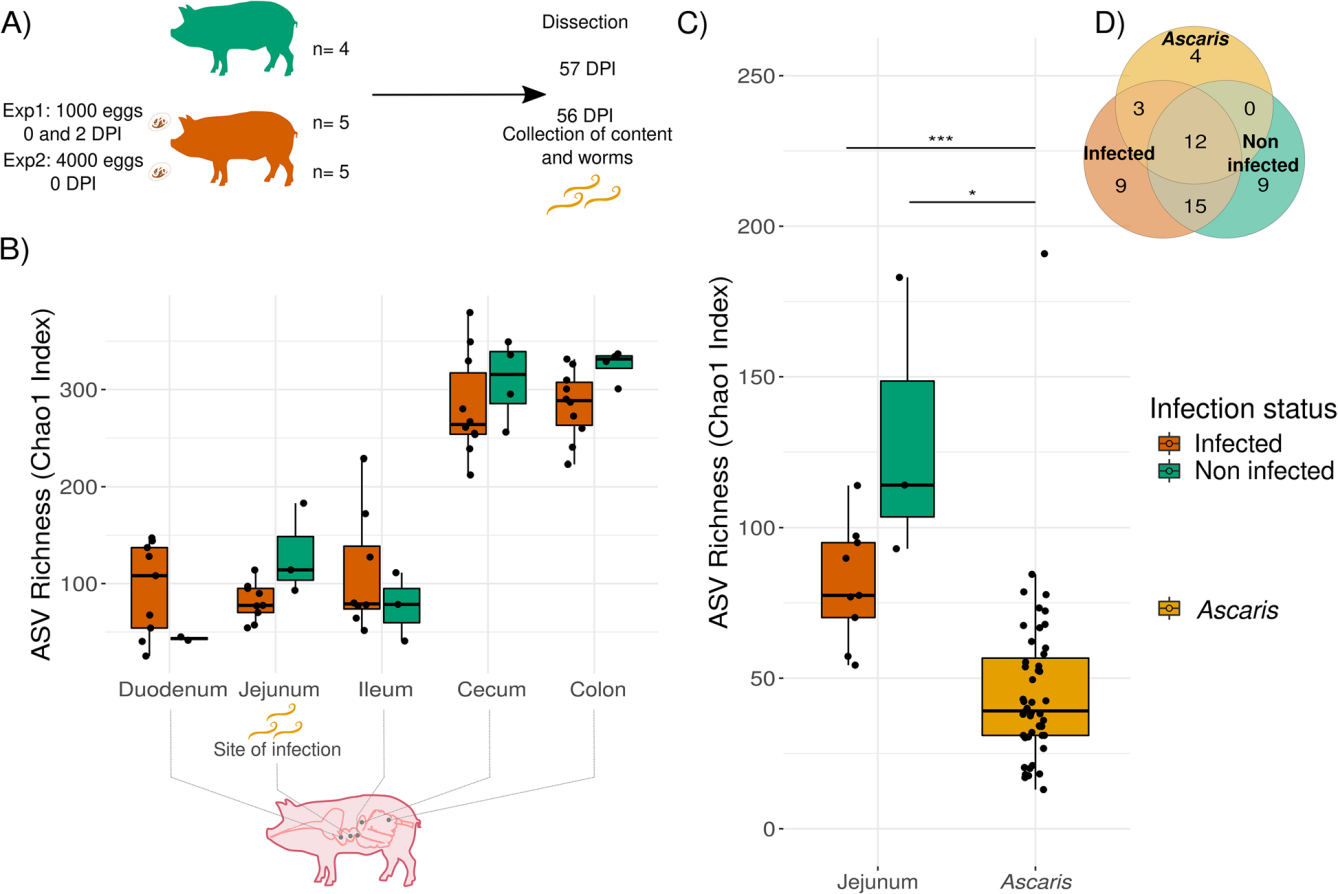
Infection reduces microbiome richness at the site of infection, and the *Ascaris* microbiome is less rich than its environment. **A** Experimental design. German Landrace pigs were infected with either two inoculums of 1000 *Ascaris* eggs (exp. 1) 2 days apart or a single inoculum of 4000 eggs (exp. 2). Four noninfected controls were also included in this study. Infection was allowed to develop to nematode patency at 56 dpi; controls were kept until 57 dpi. Porcine intestinal contents and *Ascaris* intestines were harvested for 16S microbiome sequencing. This experiment was performed in two batches. **B** Lower richness in the jejunum of infected hosts in contrast to noninfected hosts. Bacterial amplicon sequence variant (ASV) richness in the different intestinal compartments, as represented in the scheme, of infected and noninfected hosts. Each point in the box plot represents an individual pig, and the color relates to the infection status. **C***Ascaris* microbiome has significantly lower bacterial richness than both noninfected and infected host jejunum microbiome. ASV richness in the intestines of *Ascaris* worms is lower than at the site of infection (jejunum) in infected and noninfected hosts (data from **B**). Significance values: *** = *p* adjusted < 0.001, * = *p* adjusted < 0.05, Mann–Whitney *U*-(MWU) tests with Bonferroni correction for multiple testing. **D***Ascaris* share specific taxa with infected pigs at the site of infection. ASVs with relative abundance higher than 0.01% in at least 50% of the individuals were determined. Despite the significant difference in richness between jejunum and *Ascaris* microbiomes, they shared 12 highly abundant and prevalent ASVs. *Ascaris* shared three ASVs exclusively with infected pigs but none just with noninfected pigs. Jejunum microbiomes from infected and noninfected pigs shared 27 highly abundant and prevalent ASVs, and each had nine unique and specific highly abundant and prevalent ASVs

Luminal content samples were collected from several intestinal regions (duodenum, jejunum, ileum, cecum, colon) and snap-frozen in liquid nitrogen before being transferred to −80 °C until further processing. To assess the worm burden and the *A. suum* microbiome, adult worms were collected from the entire gut, counted, and morphologically separated by sex (Fig. 1A). A subset was then dissected after washing in 0.9% NaCl; their intestines harvested and snap-frozen in liquid nitrogen before storage at −80°C. Samples were homogenized with the MP FastPrep-24 homogenizer (MP Biomedicals, Eschwege, Germany), and DNA was extracted using the Macherey-Nagel NucleoSpin soil DNA extraction kit (Macherey-Nagel, Düren, Germany) according to manufacturer’s instructions.

To assess for the presence of an inherited microbiome in larvae, embryonated eggs were hatched in vitro by mechanical disruption using 5 mm glass beads and shaking. Viable larvae were purified and separated from unhatched eggs by allowing them to migrate through a cell strainer. DNA was extracted from four independent batches of *A. suum* larvae (50,000 larvae per sample) using the Macherey-Nagel NucleoSpin soil DNA extraction kit as above, with the addition of 300 g of washed and autoclaved sand (60.08 g/mol).

To estimate bacterial load in hatched L3 compared to gastrointestinal content and *Ascaris* adults, qPCR was performed using the Applied Biosystems QuantStudio 3 system (Thermo Fisher Scientific, Darmstadt, Germany) as described before [31]. In brief, amplification and detection were performed in 96-well optical plates (Applied Biosystems) with SYBR-Green (Applied Biosystems). All amplifications were performed in triplicates in a final volume of 10 μL containing 5 μL of a 10× SYBR Green PCR Master Mix including ROX as a passive reference (Applied Biosystems), 10 μM of each primer (Univ 337 F 5′-ACTCCTACGGGAGGCAGCAGT-3′ and Univ 518 R 5′-GTATTACCGCGGCTGCTGGCAC-3′), and 1.2 μL of template DNA (1.5 μg/μL).

For amplification, the standard protocol of the Applied Biosystems QuantStudio 3 system was followed, i.e., an initial cycle at 95 °C for 10 min, followed by 40 cycles at 95 °C for 15 s, and 1 min at 60 °C. To check for specificity, melting curve (Tm) analysis was performed, increasing the temperature from 60 to 95 °C at a rate of 0.2 °C per second with the continuous monitoring of fluorescence.

Standard curves for quantification consisted of 10-fold serial dilutions in the range of 10^9^–10^2^ copies of the 16S rRNA gene of the *E. coli* (Invitrogen, C404010) amplified with primers 27 F (5′-GTTTGATCCTGGCTCAG-3′) and 1492 R (5′-CGGCTACCTTGTTACGAC-3′). Copy numbers per ng DNA were calculated for each. Additionally, DNA samples from larvae were subjected to the two-step PCR method for library preparation described below.

### Library preparation and sequencing

DNA extracted from pig gastrointestinal (GI) tract contents and from *Ascaris* intestines were subjected to PCR amplification of the V3-V4 (~460 bp) hypervariable region of the 16S rRNA gene. The primers of Klindworth et al. [32] were modified to contain universal adaptor sequences for later addition of indexing barcodes as follows: forward Klin0341-19: [***ACACTGACGACATGGTTCTACA***]CCTACGGGNGGCWGCAG and reverse Klin0785_CR: [***TACGGTAGCAGAGACTTGGTCT***]GACTACHVGGGTATCTAATCC.

PCR target-specific amplification was performed with the S7 Fusion High-Fidelity DNA polymerase (Biozym Scientific GmbH, Germany) in 25 μL final volume of reaction with primers at a final concentration of 0.2 μM and 25 ng of extracted DNA under the following conditions: 95° for 30 s, followed by 35 cycles of 95° for 30 s, 60° for 30 s, 72 °C for 30 s, and a final extension of 72° for 5 min. PCR amplicons were cleaned using the magnetic beads MagBio HighPrep Clean-up kit (MagBio, USA) following the instructions of the manufacturer and eluted in 40 μL of elution buffer (10 mM Tris pH 8.5). The layout of samples over microtiter plates was randomized, including extraction controls (allowing detection of contamination during DNA extraction), non-template controls (allowing detection of contaminant DNA introduced during library preparation and PCR amplification), and a standard mock-community DNA as positive control (Zymo Research, USA). Negative and positive controls were processed and sequenced alongside the biological samples. A second PCR using 5 μL of the purified PCR products was performed employing Access Array indexing primers (Fluidigm, USA). The second PCR was run at 95° for 3 min followed by 8 cycles of 95° for 30 s, 60° for 30 s, 72° for 30 s, and a final extension at 72° for 10 min. Indexed amplicons were purified with magnetic beads and quantified using a Qubit 2.0 fluorometer with the dsDNA high sensitivity assay kit (Thermo Scientific, USA). Libraries were created by pooling each sample in equimolar concentrations. Quality and integrity of the final library were verified using the Agilent 2200 TapeStation with D1000 ScreenTapes (Agilent Technologies, USA). The pooled library was sequenced at the Berlin Center for Genomics and Biodiversity Research (BeGenDiv) on the Illumina MiSeq platform using MiSeq v2 (500 cycles) reagent kit for 2 × 250 bp paired-end reads.

### 16S sequence processing

Sequences were preprocessed to infer amplicon sequence variants (ASVs) following the pipeline of DADA2 v1.18 [33]. In brief, the raw forward and reverse reads (fastq) were truncated at base 240, as quality scores dropped at this point. Primer sequences were trimmed, and other filtering parameters were kept at default settings. Only fragments between 410 and 440 bp (expected amplicon size is ~426 bp) were further processed by removing PCR chimera sequences. Taxonomic annotation was done using the naive Bayesian classifier [34] as implemented in DADA2 with SILVA SSU database v138.1. Species were assigned for exact matches of the 16S fragments. All ASVs, metadata, and taxonomic information were compiled into a single object for further analysis using the package Phyloseq v1.22.3 [35].

### Microbiome statistical analysis

#### Data preprocessing

Further cleaning was performed as follows: (1) removing samples with zero or low read counts (less than 2000 reads), (2) discarding ASVs of nonbacterial origin or unassigned at phylum level to ensure off-target amplicon removal, and (3) removing low prevalent ASVs that do not appear more than five times in more than 10% of the samples. Samples were further analyzed according to their host (pig individual) and intestinal (duodenum, jejunum, ileum, cecum, colon) origin (Table S1). *Ascaris* microbiomes were treated as independent samples, irrespective of their eventual origin from the same host. This merged and filtered dataset was rarefied to the minimum library size and used for further alpha diversity estimation (see next section). Finally, the data was normalized by transforming ASV proportions by sample to an even depth (10^6^) for beta diversity estimations.

### Estimation of alpha and beta diversity and dominant taxa

The Chao1 index was used as a metric for richness. Alpha diversity was calculated using the package Microbiome v1.13.8 [36]. Alpha diversity was compared (1) between gastrointestinal compartments for infected and noninfected hosts, (2) between infected hosts and their parasites, and (3) between *Ascaris* from different origin or sex. Mann–Whitney *U*-(MWU) tests with Bonferroni correction for multiple testing were computed to assess significance with the package rstatix v0.7.0 [37]. Beta diversity was assessed using Bray-Curtis dissimilarity index between samples and computed using vegan v2.5-7 [38]. Comparisons of distance between individual hosts and parasite microbiomes were tested using MWU tests. Multivariate analysis was carried out using nonmetric multidimensional scaling (NMDS) with the vegan package v2.5-7 [38], also employing Bray-Curtis distance metrics as above.

Bacterial community type (enterotype) classifications were performed from the ASV abundance matrix using the Dirichlet multinomial mixture (DMM) method described in [39] and implemented in the R package *DirichletMultinomial*.

### Statistical analysis

PERMANOVA and ANOSIM tests for multivariate effect were done using the adonis and anosim function, respectively, from the vegan package v2.5-7 [38], stratified by experimental batch. PERMANOVA tests whether Bray-Curtis dissimilarity distance differs between groups and assesses marginal effects of variables, while ANOSIM tests whether distances between groups are greater than within groups. Dominant genera were defined as those with the highest relative abundance in at least one sample. The composition of ASVs belonging to those genera were then compared between different sample types (parasites, hosts, worm sexes).

Generalized linear mixed models (GLMM) were used to test whether host microbiomes at the site of infection (jejunum) and parasite microbiomes (at this site) were more similar when they came from the same host individual than from different hosts and more similar than microbiomes from other compartments. These models included only distances between microbiomes from infected hosts and worms. Bray-Curtis dissimilarity was used as the response; the sample identity of each microbiome in the paired comparison was included as a random effect to control for pseudoreplication. Paired comparisons were categorized as matching the following conditions (two predictors):

1. Both microbiomes come from the same host (yes or no).
2. Both microbiomes shared the site of infection (jejunum; yes or no; this is “yes” for *Ascaris* and the jejunum microbiome of the host, “no” for *Ascaris* and other compartments).

We also tested a statistical interaction effect between 1 and 2 to assess additivity vs. effects beyond additivity between those factors.

For each comparison, the unmatched distances were grouped as *different host*, *different compartment*, or *different individual and compartment*, respectively. Models were compared by likelihood ratio test (LRT) to determine whether each parameter was significant.

GLMM tests were also employed to test whether *Ascaris* microbiomes at the site of infection were more similar to each other when both were collected from the same pig host than when they come from different hosts and whether two *Ascaris* microbiomes were more similar when both worms had the same sex. All the models and statistical analysis are summarized by research question in Table 1.

**Table 1:** Statistical modeling

Question	Statistical approach	Response	Predictor(s)	Random effect
1) How closely does the microbiome composition of the worm resemble that of its immediate environment, the jejunum?	GLMM	Pig-*Ascaris* Bray-Curtis dissimilarity	Same host (yes, no) Location (jejunum, other compartment) Location: same host (interaction)	Individuals (pig ID and *Ascaris* ID)
2) How either host or parasite origin determine the composition of their microbiomes when only dominant microbes are taken into account?	PERMANOVA	Jejunum-*Ascaris* BC dissimilarity	Origin (host or parasite) Individual host (pig IDs)	-
3) What is the impact of the host, worm sex, and dominant bacteria on microbiome variation from pigs and *Ascaris*?	PERMANOVA	Pig-*Ascaris* BC dissimilarity	Host (pig IDs) Worm sex (male, female) Dominant bacteria (*Clostridium sensu stricto* 1 *Lactobacillus*, *Escherichia-Shigella*, *Prevotella*, *Streptococcus*, and *Romboutsia*)	-
4) What is the impact of the host of origin and worm sex on microbiome variation among *Ascaris* individuals?	GLMM	*Ascaris*-*Ascaris* BC dissimilarity	Same host (yes, no) Same sex (yes, no)	Individuals (*Ascaris* IDs)
5) Is the infection status a relevant factor driving the differences in microbial composition between host and parasites?	GLMM	Pig-pig BC dissimilarity	Same host (yes, no) Same compartment (yes, no) Same infection status (yes, no)	Individuals (pig IDs)

### Impact of dominant taxa on jejunum-*Ascaris* microbiome

To investigate whether composition variation between the site of infection and parasites is driven by the most dominant bacteria (see section above, 'Estimation of alpha and beta diversity and dominant taxa'). Jejunum-*Ascaris* microbes were analyzed using the microbial (ASV level) composition restricted to dominant taxa. Bray-Curtis dissimilarity was estimated, and further PERMANOVA and ANOSIM analysis was done using individual pig and host or parasite assignment as predictors.

### Identification of differentially abundant bacterial group microbiomes

ASV enrichment was tested as a function of (1) host jejunum against *Ascaris* microbiomes and (2) *Ascaris* female against male microbiomes. DESeq2 package v1.30.1 was used for the assessment; this pipeline uses negative binomial distribution models that account for differences in library sizes to test for differential abundance between testing conditions using the Wald statistics test [39]. Raw counts were used, and the pipeline ran under default settings. The *q*-values were calculated with the Benjamini-Hochberg procedure [40] to correct *p*-values and control for false discovery rates. All significant ASVs were additionally checked using NCBI BLAST searches against the NCBI nr database to confirm their identity.

## Results

The parasite and host microbiome sequencing data contained 3,004,508 total reads with an average of 12,677 reads/sample, ranging between 2090 and 57,121 reads. A total of 7934 amplicon sequence variants (ASV) were derived, with an average of 125 ASVs/sample. A total of 172 genera were detected across all samples. We found no evidence of bacterial DNA in larval samples (Fig. S1) and thus focused our analyses on adult parasites and host contents.

### The *Ascaris* microbiome is less rich than that of its porcine host

In order to decipher the microbiome of the parasite gut we first asked, is an *Ascaris* infection beneficial or detrimental for the microbial diversity of the host gut, and does *Ascaris* influence the host microbiome along the entire gut? To locate the effects of *Ascaris* infection on the host microbiota, we assessed the alpha diversity and compared the richness among different intestinal compartments of infected and noninfected pigs. We observed a general and progressive increase in the richness from the small intestinal compartments further down the gut to the colon, independent of the infection status. Interestingly, a notably lower richness was detected in the jejunum of infected hosts in contrast to noninfected hosts, independently from the batch of the experiment (Fig. 1B). The extent of richness decrease at the site of infection was not correlated with individual worm burden (Fig. S2), suggesting a decrease in resident host bacteria between microbiomes of infected and noninfected hosts independent of infection intensity.

Next, we analyzed the microbiome richness of the parasites and compared it to the microbiome richness of the host (from Fig. 1B). At the site of infection in the jejunum, we observed that resident *Ascaris* worms presented a significantly lower bacterial richness than both noninfected and infected host intestines (Fig. 1C), implying that the *Ascaris* gut microbiome is less diverse compared to its host. This observation prompted us to determine whether *Ascaris* and its host share specific bacteria and which groups characterize each microbiome.

The search for the core microbiome involves determining which taxa, if any, are shared among two or more microbial communities. To infer whether hosts and parasites have a core microbiome, we aimed to explore the shared highly abundant and prevalent ASVs between noninfected and infected pigs as well as *Ascaris* worms. We defined the highly abundant and prevalent ASVs for jejuna from noninfected and infected pigs and *Ascaris* (microbial taxa shared by most of the different studied microbiomes) as the group of ASVs with relative abundance higher than 0.01% in at least 50% of the individuals. Those shared ASVs between sample types were considered as “core microbiome” (Fig. 1D; Table S2). Infected jejuna in our study exhibited a total of 39 highly abundant and prevalent ASVs, while the noninfected jejunum presented 36 highly abundant and prevalent ASVs. Infected and noninfected host jejunum microbiomes shared 27 highly abundant and prevalent ASVs; however, while 12 were also in *Ascaris* microbiomes, 15 were exclusively detected in jejunum microbiomes. Exclusively shared microbes between infected and noninfected host microbiomes suggest a set of taxa of a jejunum core microbiome. In contrast, *Ascaris* microbiomes had just four unique ASVs and shared three exclusively with infected pigs (ASV4 — *Escherichia-Shigella*; ASV29 — *Lactobacillus pontis*, and ASV40 — *Lactobacillus*) but none just with noninfected pigs.

In conclusion, an *Ascaris* infection leads to a loss of microbial diversity of the host gut, though only at the site of *Ascaris* infection in the small intestine. The parasite microbiomes differ drastically in diversity from their host environments, being less diverse while sharing specific taxa with infected pigs at the site of infection.

### *Ascaris* microbiomes are similar to their host microbiome at the site of infection

**Fig. 2:**
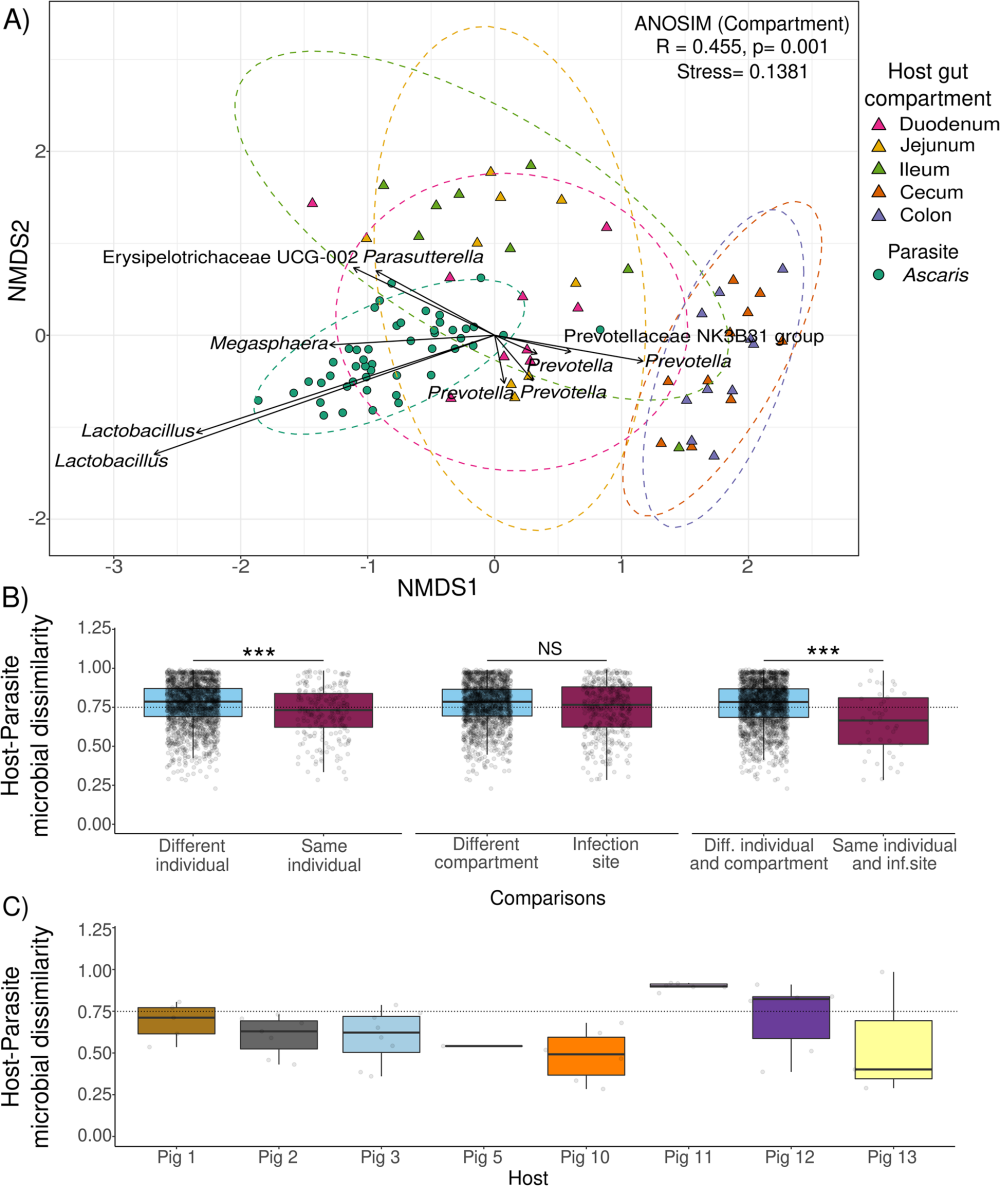
Characterization of microbial communities between hosts and *Ascaris* worms **A***Ascaris* microbiome composition is closer to the upper gastrointestinal tract microbiome than to the colon and cecum. Nonmetric multidimensional scaling (NMDS) showing differences in microbial composition among gastrointestinal compartments from infected individuals: duodenum, jejunum and ileum (upper GI tract), cecum and colon (lower GI tract), and *Ascaris* worms. Each triangle in the graph represents an infected individual, circles represent individual *Ascaris*, and distances between points are proportional to their biological dissimilarity, calculated with the Bray-Curtis index. The color of the points and the dotted lines surrounding them represents the clusters by compartment. NMDS shows general proximity among *Ascaris* microbiomes and those from upper GI tract compartments, particularly to jejunum and duodenum, the jejunum being the distinct site of infection. Arrows represent the top ASVs (genus level) linked to NMDS axes; their length reflects the relative importance of the ASV on the respective axes. **B** Host and parasite microbiome compositions are closer when both come from the same individual and the infection site. Dissimilarity among bacterial communities derived from *Ascaris* to those from the infected hosts is shown. Host and parasite microbiomes were significantly more similar when they came from the same host individual (LRT: *χ*^2^ = 52.349, *df* = 1, *p* < 0.001; left). Microbiomes from the shared gut compartment (host microbiomes from the jejunum and those of *Ascaris*) are only more similar when they additionally come from the same host individual (LRT: *χ*^2^ = 33.821, *df* = 1, *p* < 0.001; right). If the host individual was not taken into account, only the kind of compartment (jejunum) did not significantly explain microbiome similarity (LRT: *χ*^2^ = 0.441, *df* = 1, *p* = 0.507; middle). The dashed line highlights that the median microbial dissimilarity in host-parasite microbiomes from *different individual*, *different compartment*, and *different individual and compartment* is above 0.75. **C** The majority of *Ascaris* microbiomes are similar to their host’s jejunum microbiome. Bray-Curtis dissimilarity values to assess the similarity between worms within the same hosts. In six out of eight pigs, the jejunum-*Ascaris* microbial dissimilarity has a median below the overall median of host-parasite dissimilarity (0.75; dashed line). This is a graphical representation of the significantly higher similarity between host-parasite pairs from the same host than host-parasite pairs from different hosts (GLMM; Table 2). This also points to residual variability in the similarities, so that (only) few worms’ microbiomes are still less similar to their own hosts microbiome than to that of the other hosts. The closer the values are to zero, the more similar the microbiome compositions

Our next question was where is the parasite microbiome derived from? Having observed a less diverse worm microbiome, we now assessed how closely the microbiome composition of the worm resembles that of its immediate environment, the jejunum. To compare microbiome composition between intestinal compartments from infected hosts and their worms, we used permutational analysis of variance (PERMANOVA) based on Bray-Curtis dissimilarities. Nonmetric multidimensional scaling (NMDS) analysis shows that *Ascaris* microbiomes cluster closer to microbiomes from the upper intestinal compartments (Fig. 2A). In particular, the *Ascaris* microbiome is more similar to the microbiome of the jejunum and duodenum and more different to the colon microbiome. ANOSIM results showed that both host-parasite differentiation and intestinal compartment are the significantly influential parameters explaining the clustering of the samples (*ANOSIM*_compartment_: *R* = 0.455, *p* < 0.01; *ANOSIM*_host-parasite_: *R* = 0.378, *p* < 0.01). The differentiation between the host or parasite microbiome (PERMANOVA, *R*^2^ = 0.157, *p* < 0.01) and intestinal compartment (PERMANOVA, *R*^2^ = 0.137, *p* < 0.01) explained almost 30% of the variation (Table S4), suggesting a higher impact from the worms’ microbiomes in the clustering than the infection status per se. In addition, enterotype clustering and the best Dirichlet multinomial mixture model (DMM, *π* = 0.284, *θ* = 247.873, *k* = 2, Laplace: 175799.8 BIC: 198685.3 AIC: 188431.3) confirmed the similarity of *Ascaris* microbial communities to the upper GI tract by classifying the samples into two enterotypes (Fig. S3). The first enterotype contained all samples from the upper gastrointestinal tract (GI; duodenum, jejunum, and ileum) and most of the *Ascaris* microbiomes (*N* = 46/47), while a second enterotype included all lower GI samples and one *Ascaris* sample.

To shed light on the origin of *Ascaris* microbiomes, we tested whether the parasite microbiome composition was more similar to a specific gut compartment (the jejunum, the infection site) and whether an individual *Ascaris* microbiome was more similar to that of the individually infected host than to that of other hosts. To test this, we compared the host-parasite microbiome dissimilarity (Bray-Curtis). We observed that host and parasite microbiomes were significantly more similar when they came from the same host individual (LRT: *χ*^2^ = 52.349, *df* = 1, *p* < 0.001; Table 2, Fig. 2B, left). In addition, microbiomes from the shared gut compartment (host microbiomes from the jejunum and those of *Ascaris*) are only more similar when they additionally come from the same host individual (LRT: *χ*^2^ = 33.821, *df* = 1, *p* < 0.001; Table 2, Fig. 2B, right). If the host individual was not taken into account, only the kind of compartment (jejunum) did not significantly explain microbiome similarity (LRT: *χ*^2^ = 0.441, *df* = 1, *p* = 0.507; Table 2, Fig. 2B, middle).

**Table 2:** GLMM to assess microbial dissimilarity among host-parasite microbiomes

	Estimate	SE	***t***-value	Chisq	***P***-value
**Model: host-parasite microbial dissimilarity**					
Intercept	0.7737	0.0188	41.201	-	-
***Same individual***	−0.0351	0.0060	−5.873	52.349	< 0.001**
Same site of infection	−0.0221	0.0332	−0.665	0.441	0.507
***Same individual and site of infection*(statistical interaction effect)**	−0.0793	0.0136	−5.840	33.821	< 0.001***

*SE* standard error, *t-value t*-test statistic, *Chisq* likelihood ratio chi-squared statistic and *p-*value

Significance codes, ***0.001, **0.01, *0.05

Considering that host-parasite microbiome similarity increases when both come from the infection site within the same host, we specifically analyzed the bacterial composition of the jejunum (site of infection) and worm microbiomes by the individual origin of the sample (individual pig) to determine whether the majority of worms within the same environment showed the same degree of similarity in all hosts. We observed that worm and jejunum microbiomes from the same host presented a trend of high similarity (values closer to zero, median below 0.75) for six out of eight pigs (Fig. 2C), suggesting a general close similarity of worm microbiome with the microbiome of their respective host at the site of infection (values closer to one). Most (but not all) individual worms are more similar to their individual host than the average similarity between host-parasite pairs. The discrepancy of few individual worm microbiomes showing lower similarity to their hosts might be attributed to differences in their genotype or developmental stage, indicating worm microbiome individuality. Pig 4 and pig 14 were not included in this analysis as they lack either *Ascaris* or jejunum microbiome information, respectively. We found that the individual pig of origin explains 45% of variation (PERMANOVA, *R*^2^ = 0.454, *p* < 0.01; ANOSIM, *R* = 0.431, *p* < 0.01) while being jejunum or worm (host-parasite parameter) accounts for less than 10% (PERMANOVA, *R*^2^ = 0.093, *p* < 0.01; ANOSIM, *R* = 0.463, *p* < 0.01) (Table S4), suggesting a close proximity of both jejunum and *Ascaris* microbiomes within the same individual.

In addition, for the entire GI tract, nonmetric multidimensional scaling (NDMS) shows the differences in bacterial composition among GI compartments, but the derived configuration is not necessarily linked to the infection status (Fig. S4). Our data show a clear differentiation between the upper GI tract compartments (duodenum, jejunum, and ileum) and compartments of the large intestine (caecum and colon) driven by some ASVs annotated as *Anaerosporobacter*, *Lactobacillus*, and *Parasutterella* but not by infection status (Fig. S4A). Within the same intestinal compartment, the Bray-Curtis distance between infected and uninfected hosts does not significantly differ from that between infected or between uninfected hosts. This confirms the minor effect of infection status on microbial composition (Fig. S3B). We observed that overall variation of the GI microbiome composition varies with intestinal compartment (*R*^2^ = 0.314, *p* < 0.01). Overall, the infection status (*R*^2^ = 0.069, *p* < 0.01) and the individual pig (*R*^2^ = 0.246, *p* < 0.01) had significant but smaller effects (Tables S5 and S6). Analysis of similarity (ANOSIM) confirmed this effect of compartment (*R* = 0.475, *p* < 0.01), infection status, (*R* = 0.066, *p* < 0.01), and individual host (*R* = 0.231, *p* < 0.01) on microbiome dissimilarities. Despite the apparent decrease in richness between infected and noninfected pigs, there is a stronger effect of the compartment on the microbial composition than the effect of infection status.

Taken together, our data show that the less diverse *Ascaris* microbiome is similar to its immediate host microbial environment, rather than containing a random subset of host microbiomes.

### Differences in microbial composition are driven by dominant bacteria

**Fig. 3:**
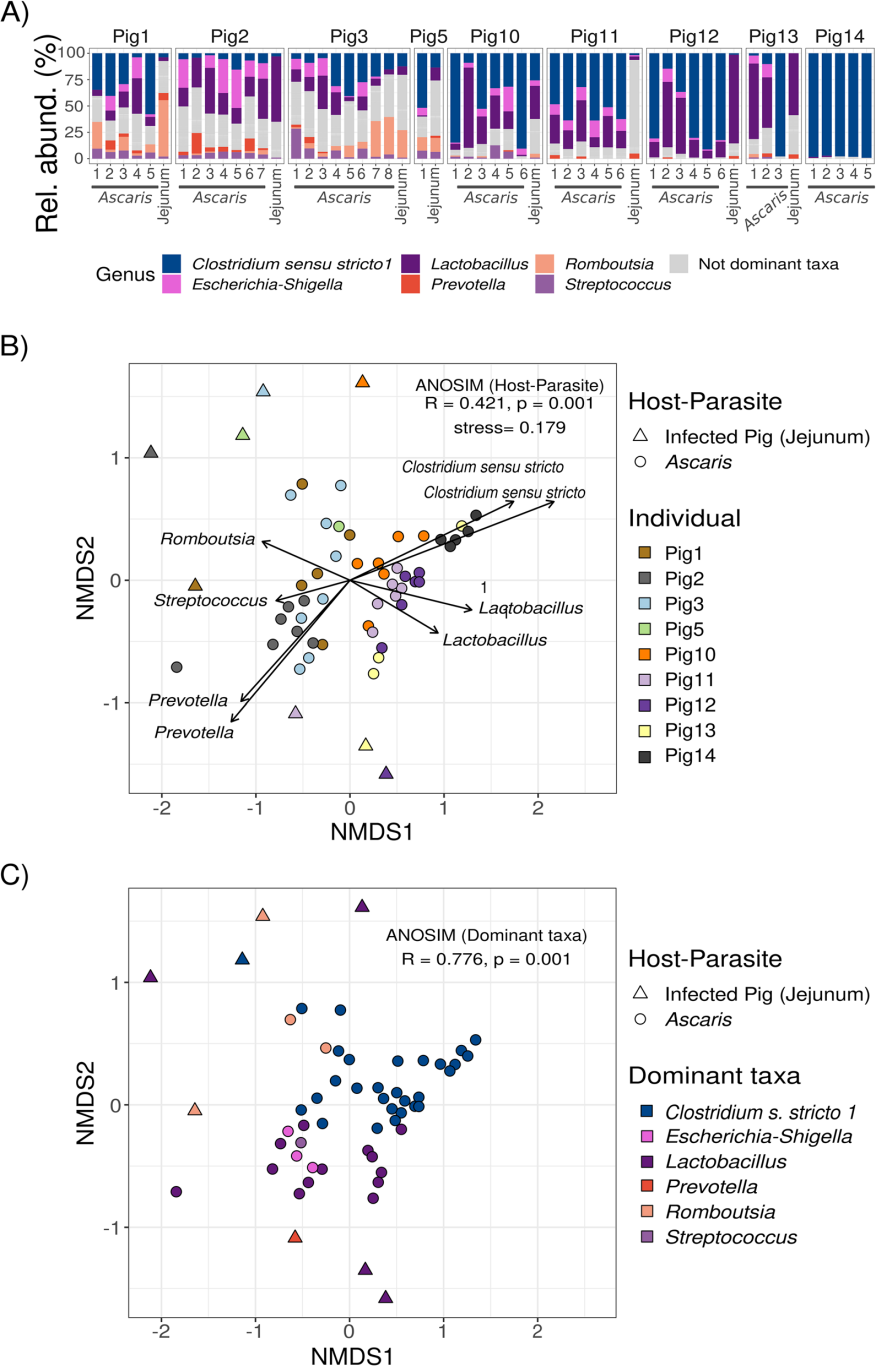
Host of origin and dominant taxa are the main drivers of differences in microbial composition among host and *Ascaris*. **A** Bacterial composition in *Ascaris* and its host jejunum microbiome. Composition of worms and host-associated microbiomes do not show a clear pattern of relative abundance linked to the host; nevertheless, we observed six dominant bacteria represented by *Clostridium sensu stricto* 1, *Escherichia-Shigella*, *Lactobacillus*, *Prevotella*, *Romboutsia*, and *Streptococcus* as bacteria with the higher relative abundance, dominating the communities. All nondominant taxa were shown as a single group. **B** The similarity of *Ascaris* and jejunum microbiome compositions is determined by the individual of origin. Microbial composition restricted to the six dominant taxa among host jejunum, and the microbiome from *Ascaris* worms infecting them shows differences detectable via nonmetric multidimensional scaling (NMDS). Individual pigs explain most of the variation (45%) PERMANOVA, *R*^2^ = 0.454, *p* < 0.01; ANOSIM, *R* = 0.421, *p* < 0.01). Arrows represent the top ASVs (genus level) linked to NMDS axes; their length reflects the relative importance of the ASV on the respective axes. **C** The jejunum and *Ascaris* samples clustered based on their dominant bacteria. Detecting dominant bacteria (most dominant genus within each community) showed worms and jejunum belonging to the same dominant bacteria cluster (ANOSIM, *R* = 0.776, *p* < 0.01). Together with (**B**), it was possible to confirm that the individual host and the dominant bacteria are the most relevant factors linked to the clustering of the samples

We now asked, is there a detectable core microbiota in the parasite gut? After finding the worm microbiome to be highly similar to the microbiome of the individual host at the site of infection, we aimed to investigate whether this similarity between the parasite and its host microbiome is driven by ASVs belonging to the most dominant bacterial genera. For this purpose, we defined the dominant bacteria as the taxa at genus level with the highest relative abundance within any one of the microbial communities. Six genera are the most dominant in jejunum and *Ascaris* microbiomes: *Clostridium sensu stricto* 1 (29 samples), *Lactobacillus* (17 samples), *Escherichia-Shigella* (3 samples), *Romboutsia* (4 samples), *Prevotella* (1 sample), and *Streptococcus* (1 sample). The most dominant bacterial genus in the jejunum is *Lactobacillus* (in 4 out of 8 pigs). *Ascaris* microbiomes derived from those pigs have microbiomes dominated by either *Lactobacillus* or *Clostridium sensu stricto* 1 (13 and 28 worms, respectively) (Fig. 3A). *Escherichia*-*Shigella* and *Streptococcus* are dominant in just three and one *Ascaris* microbiome, respectively. *Prevotella* dominates in just one jejunum microbiome, while *Romboutsia* dominates in each of two *Ascaris* and pig samples.

To determine the impact of the dominant bacteria on microbiome variation, we restricted jejunum and *Ascaris* compositions to ASVs taxonomically assigned to the six dominant genera observed in 3A. We observed that 45% of the variation in microbiomes with dominant-restricted compositions was explained by individuals of origin, linked to the distribution of *Ascaris* samples closer to jejunum from the same pig rather than to a different host (Fig. 3B). The observed variation explained by individuals in the dominant-restricted compositions did not increase compared to the whole bacteria composition. This might point to abundance-independent differences between host and parasite microbiomes: either highly or lowly abundant taxa can make the difference between hosts. The microbiome subset in worms is derived from their respective individual hosts without preference for either lowly or highly abundant (dominant) bacteria, but dominant bacteria are primary drivers of microbiome composition due to their sheer abundance. Additionally, the distribution of samples along NMDS axes relates to the relative importance of the ASV assigned to *Clostridium sensu stricto* 1, *Lactobacillus*, *Prevotella*, *Streptococcus*, and *Romboutsia* (arrows in Fig. 3B), while the origin of the microbiome, either being jejunum or worm (Host-Parasite parameter), explains less than 10% of the variation (PERMANOVA, *R*^2^ = 0.454, *p* < 0.01; ANOSIM, *R* = 0.397, *p* < 0.01) (Table 3) as when including nondominant taxa (Table S4).

**Table 3:** Permutational analysis of variance for dominant bacterial taxa composition in jejunum and *Ascaris* from infected pigs

	Df	Sums of squares	Mean sqs.	***F***-model	***R***^**2**^	Pr (> ***F***)
*Host or parasite*	1	1.0203	1.0203	9.282	0.093	0.001***
*Host individual*	8	4.9599	0.620	5.640	0.454	0.001***
**Residuals**	45	4.9467	0.110	-	0.453	-
**Total**	54	10.9269	-	-	1.000	-

*Df* degrees of freedom, *F-model pseudo*-*F*-test statistic, *R*^*2*^ variance explained and *p-*value based on 999 permutations

Significance codes, ***0.001, **0.01, *0.05

Lastly, each dominant-restricted microbiome was categorized based on the genus with the highest relative abundance within each sample. By indicating the highest dominant taxa in the microbiomes, we confirmed that jejunum and *Ascaris* clustering is driven by the individual host and *Clostridium sensu stricto* 1 *Lactobacillus*, *Escherichia-Shigella*, *Prevotella*, *Streptococcus*, and *Romboutsia*. A comparison of Bray-Curtis distances between samples among dominant groups confirmed the significantly high dissimilarity (ANOSIM, *R* = 0.776, *p* < 0.01). When matched to individual identification from Fig. 3B, the jejunum microbiome from pigs 2, 3, 5, 12, and 13 shared the dominant taxa with at least one worm microbiome collected from them, and in three pigs (1, 10, and 11), this was not the case (Fig. 3C).

Taken together, these data indicate that the host of origin and dominant taxa of the infected host respectively are the main drivers of the differences in microbial composition among *Ascaris* samples.

### Parasite sex does not considerably impact microbiome composition in worms

**Fig. 4:**
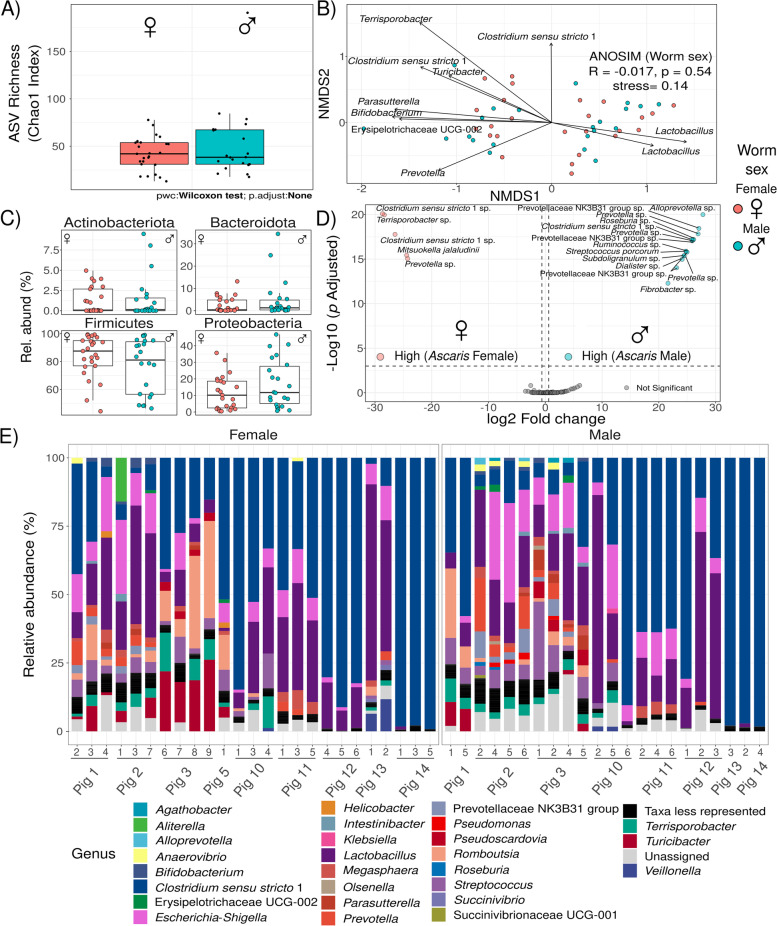
Characterization of microbial communities in *Ascaris* worms. **A** Female and male *Ascaris* microbiomes do not differ in richness. ASV richness from *Ascaris* worms was not linked to the sex of worms. The lack of sex difference in ASV richness was independent of experimental batch effects. **B***Ascaris* microbiome composition is not determined by worm sex. Worm sex plays a minor and nonsignificant role in clustering of worm microbiomes (*PERMANOVA*_sex_: *R*^2^ = 0.009, *p* > 0.05; *ANOSIM*_Sex_: *R* = 0.091, *p* > 0.05) compared to the dominant bacteria or the host of origin. **C** The abundance of main phyla in *Ascaris* microbiomes is not different between worm sexes. A nonsignificant difference in abundance of the main bacterial phyla between *Ascaris* worms of both sexes was detected. However, only a trend for Firmicutes abundance in females and Proteobacteria abundance in males was detected. **D** Differential bacterial ASVs in female and male *Ascaris*. Despite there being no overall community differences, particular bacterial taxa (ASVs) were differentially abundant between male and female worms. Each point depicts log_2_ fold differential abundance values (x-axis) and −log_10_ of the adjusted *p*-values (odds ratio). Values > 0 represent enrichment of the 15 ASVs in the males compared to females which included *Bifidobacterium* and *Lactobacillus*. Values < 0 reflect differential abundance of 5 ASVs in the females compared to males, including *Clostridium sensu stricto* 1 and members from the family Prevotellaceae. **E** Microbial composition at genus level of worms by sex. Relative abundance at genus level is presented for each individual worm collected from infected pigs. Genus with less than 1% relative abundance are binned as *Taxa less represented*

The next major question was as follows: are gut microbes distinct in female parasites compared to males? Female *Ascaris* worms are significantly larger than males, and individual females release over 200,000 eggs per day [41]. As female worms may depend on particular microbial metabolites for their excessive reproduction, we investigated whether the sex of worms is associated with differences in the diversity or specific composition of microbes. We compared the alpha diversity (ASV richness) of *Ascaris* worms depending on their sex. We did not observe differences in ASV richness linked to the sex of the worms (Fig. 4A). The lack of sex difference with regard to the bacterial composition in worms was independent of the experimental batch effects, as for the richness in infected and noninfected pigs (Fig. 1B). We compared microbial composition between worms from different sexes. Our data indicate that sex plays a minor role as a driver of the bacterial composition in the worm and did not achieve significance (*PERMANOVA*_sex_: *R*^2^ = 0.009, *p* > 0.05; *ANOSIM*_Sex_: *R* = 0.091, *p* > 0.05) (Fig. 4B), while the dominant bacteria of the individual host is the most relevant driver of the bacterial composition in the worm (*PERMANOVA*_dominant bacteria_: *R*^2^ = 0.244, *p* < 0.01; *ANOSIM*_dominant bacteria_: *R* = 0.759, *p* < 0.01; *PERMANOVA*_Individual_: *R*^2^ = 0.532, *ANOSIM*_Individual_: *R* = 0.485, *p* < 0.01) (Table 4). To confirm the drivers in *Ascaris* bacterial composition, we compared parasite-parasite Bray-Curtis microbial dissimilarity using a GLMM approach. We confirmed that coming from the same host (LRT: *χ*^2^ = 113.61, *df* = 1, *p* < 0.001) better explained microbiome composition proximity between *Ascaris* worms (Table 5). Microbiomes from worms of the same sex are not more similar than those of worms with different sexes (LRT: *χ*^2^ = 0.105, *df* = 1, *p* = 0.746).

**Table 4:** Permutational analysis of variance for bacterial taxa composition in *Ascaris* from infected pigs

	Df	Sums of squares	Mean sqs.	***F***-model	***R***^**2**^	Pr (> ***F***)
*Host individual*	8	4.8524	0.607	10.209	0.532	0.001***
*Worm sex*	1	0.0811	0.081	1.364	0.009	0.225
*Dominant bacteria*	4	2.2273	0.557	9.372	0.244	0.001***
**Residuals**	33	1.9606	0.059	-	0.215	-
**Total**	46	9.1214	-	-	1.000	-

*Df* degrees of freedom, *F-model pseudo*-*F*-test statistic, *R*^*2*^ variance explained and *p-*value based on 999 permutations

Signif. codes, 0***, 0.001**, 0.01*, 0.05‘.’, 0.1‘ ’, 1

**Table 5:** GLMM to assess microbial dissimilarity proximity among parasite-parasite microbiomes

	Estimate	SE	***t***-value	Chisq	***P***-value
**Model: parasite-parasite microbial dissimilarity**					
Intercept	0.6085	0.0205	29.703	-	-
***Same host***	−0.2114	0.0193	−10.964	113.61	< 0.001***
Same sex	0.0033	0.0103	0.324	0.105	0.746

*SE* standard error, *t-value t*-test statistic, *Chisq* likelihood ratio chi-squared statistic and *p*-value

Significance codes: ***0.001, **0.01, *0.05

Having found similar ASV richness between male and female worms, we sought to assess compositional differences between worms of different sexes. We did not observe a difference in the abundance of the four major phyla: Actinobacteoriota, Bactoriodota, Firmicutes, and Proteobacteria between female and male worms (Fig. 4C). Despite there being no overall community level differences between worm sexes (alpha and beta diversity not significantly altered), we found a few ASVs differentially abundant between males and females. Male worms presented 15 differentially abundant ASVs compared to females that presented five ASVs (Fig. 4D; Table S7). Males have *Prevotella* or members of the family Prevotellaceae as the most represented ASVs, in contrast to females in which the majority of the enriched ASVs belong to *Clostridium sensu stricto* 1. The few taxa showing differences do not impact the overall composition, as observed in Fig. 4A–C, if they are not relevant to the microbiome structure (keystones). Microbial composition of worms by sex (Fig. 4E) generally shows similarly abundant genera of bacteria; *Clostridium sensu stricto* 1, *Escherichia-Shigella*, *Lactobacillus*, *Prevotella*, *Romboutsia*, and *Streptococcus* have the highest relative abundance compared to the rest and dominate the communities in both female and male worms. Given the lack of sex effect in the overall bacterial composition of the worm intestines, the few differentially abundant ASVs can probably not be linked to any worm physiological function. In conclusion, these results further highlight the importance of the host of origin and dominant host genera as essential determinants of the *Ascaris* microbiome.

### Different bacterial groups are enriched between pigs and *Ascaris* microbiomes

**Fig. 5:**
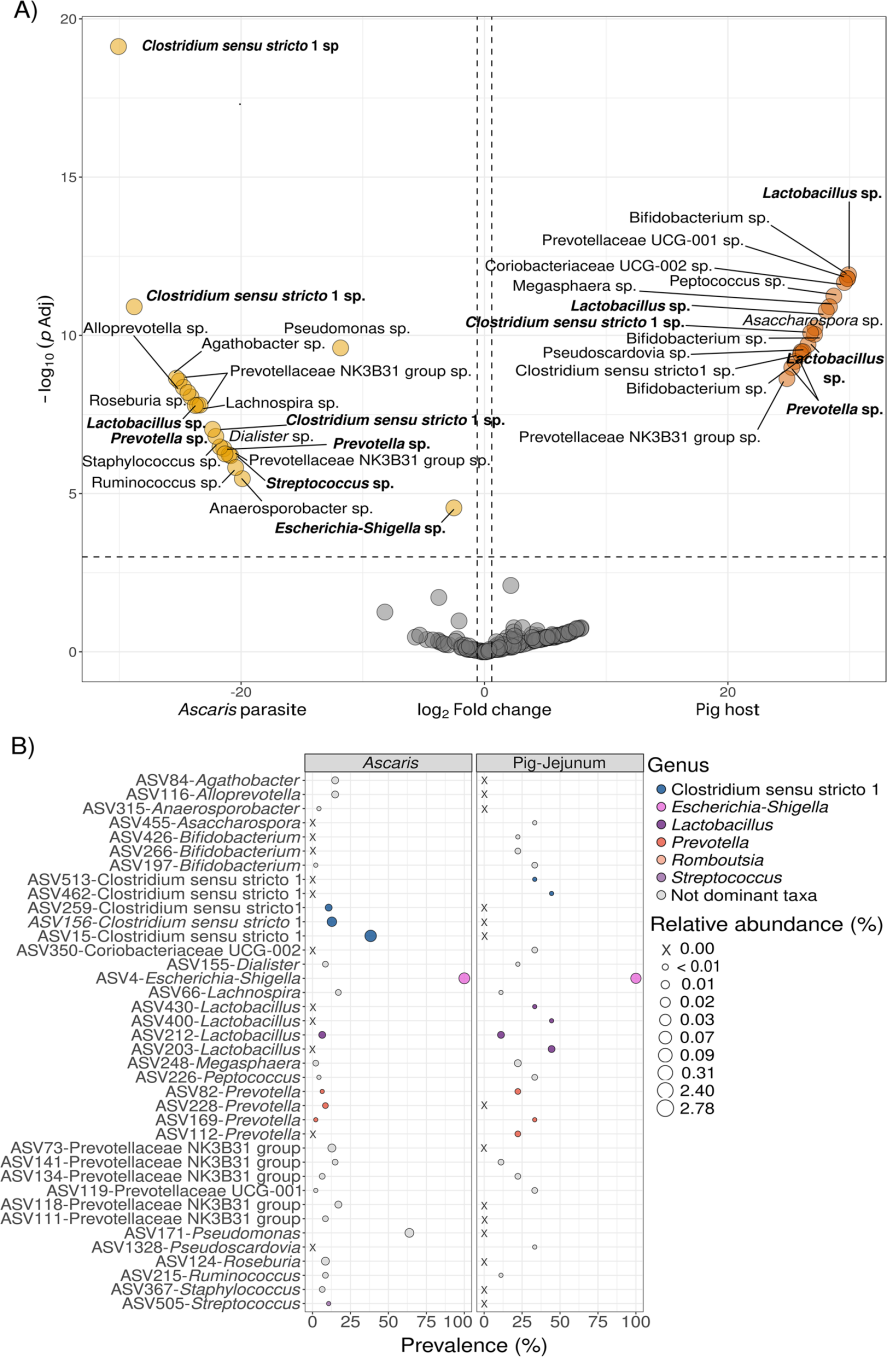
Bacterial groups are enriched between pigs and *Ascaris* microbiomes. **A** Differentially abundant bacteria between *Ascaris* and jejunum. Significantly enriched ASVs stratified by host and parasite microbiomes (infected jejunum and *Ascaris* from infected individuals). Each point represents log_2_ fold enrichment values. Values > 0 represent enrichment of the ASV in the pig jejunum microbiome compared to the *Ascaris* microbiome from each comparison. Values < 0 reflect enrichment of the taxon in the *Ascaris* microbiome compared to the pig jejunum microbiome. Taxa in bold indicate ASVs that belong to dominant genera. **B** Out of the differentially abundant bacteria, specific ASVs are exclusive to *Ascaris* or host microbiomes. General prevalence either in *Ascaris* microbiome or pig jejunum microbiome of all significant differentially abundant ASVs in (**A**). Size of the circles indicates the relative abundance in the respective microbiomes; crosses (X) indicate the absent ASVs in either one or the other microbiome type. Those ASVs belonging to dominant taxa are colored accordingly

Finally, we asked, do the intestines of the parasites show a depletion or enrichment of specific bacterial species? Having ascertained that dominant ASVs at the site of infection in the host of origin serve as the primary determinants of the *Ascaris* microbiome, we now characterized the constituents of the *Ascaris* microbiome in relation to the jejunum. To test whether specific bacteria are enriched or depleted in the microbiomes of worms compared to their hosts, we performed an analysis of differential abundance. In jejunum samples from infected pigs, 17 ASVs were enriched, compared to 21 ASVs enriched in *Ascaris* (Fig. 5A, Table S8). In jejunum microbiomes, those include seven ASVs belonging to the dominant taxa *Lactobacillus* (ASV203, ASV400, and ASV430), *Clostridium sensu stricto* 1 (ASV462, ASV513), and *Prevotella* (ASV112 and ASV169) and ten more to nondominant taxa *Bifidobacterium* (ASV197, ASV266, and ASV426), *Peptococcus* (ASV226), *Pseudoscardovia* (ASV1328), *Asaccharospora* (ASV455), and *Megasphaera* (ASV248) and from the families Prevotellaceae (ASV119 and ASV134) and Coriobacteriaceae (ASV350). In *Ascaris* gut microbiomes, eight ASVs belonged to the dominant taxa *Clostridium sensu stricto* 1 (ASV15, ASV156, and ASV259), *Prevotella* (ASV82, ASV228), *Lactobacillus* (ASV212), *Streptococcus* (ASV505), and *Escherichia*-*Shigella* (ASV4) and 13 more to nondominant taxa mainly from the family Prevotellaceae (ASV73, ASV111, ASV118, ASV141) and to the genus *Alloprevotella* (ASV116), *Agathobacter* (ASV84), *Anaerosporobacter* (ASV315), *Dialister* (ASV155), *Lachnospira* (ASV66), *Pseudomonas* (ASV171), *Roseburia* (ASV124), *Ruminococcus* (ASV215), and *Staphylococcus* (ASV367).

The distribution of *p*-values and fold changes in abundance generates clear, distinct microbe groups for hosts and parasites (Fig. 5A). Despite the microbial composition being driven by some dominant genera like *Lactobacillus*, *Prevotella*, and *Clostridium sensu stricto* 1, less abundant microbes (relative abundance below 0.1%) primarily characterize each of the communities (Fig. 5B). *Alloprevotella*, *Agathobacter*, *Anaerosporobacter*, *Dialister*, *Lachnospira*, *Roseburia*, *Ruminococcus*, and *Staphylococcus* are low abundant genera (less than 0.09% relative abundance) but distinctive in *Ascaris* microbiomes compared to host jejunum microbiota. In addition, *Pseudomonas* is an example of an exclusive, highly prevalent (> 50% prevalence) microbe in the *Ascaris* gut microbiome. The ASV belonging to *Escherichia-Shigella* was prevalent in both community types, slightly differentially abundant but significant in *Ascaris* microbiomes. Interestingly, jejunum and *Ascaris* microbiomes had differential and exclusive ASVs from *Clostridium sensu stricto* 1, *Lactobacillus*, and *Prevotella*, suggesting characteristic microbes in worm microbial communities.

**Fig. 6:**
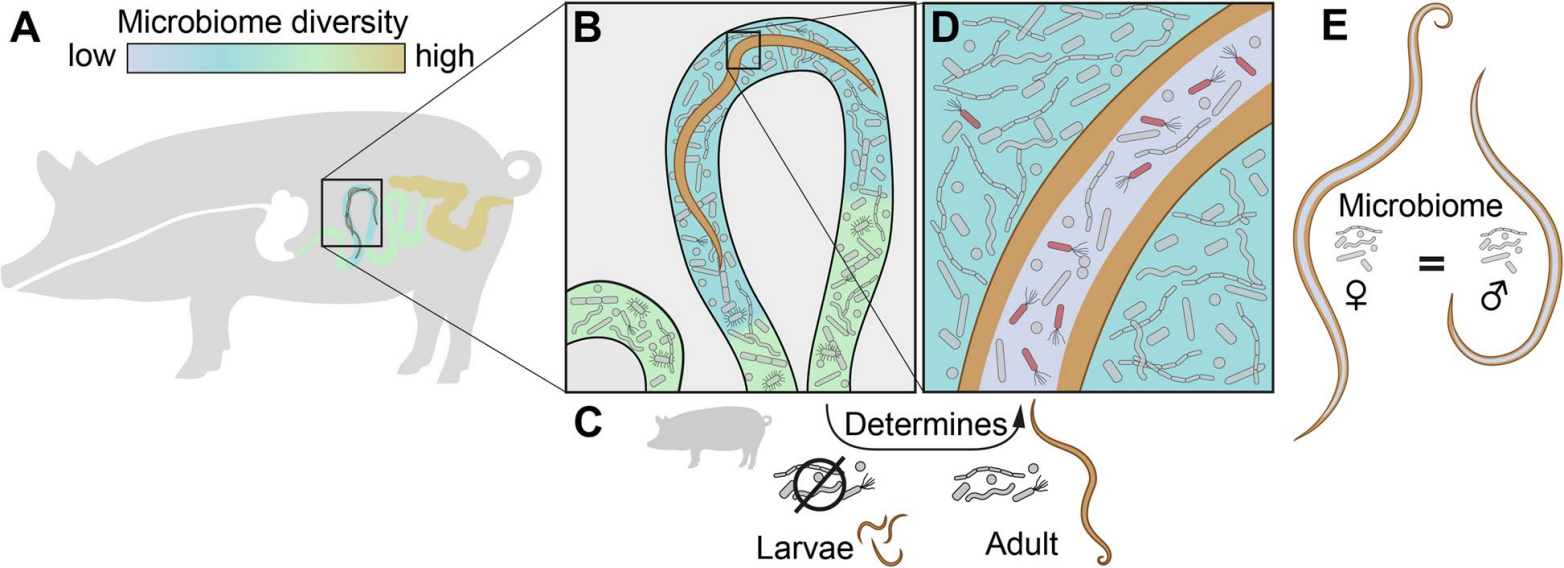
**A** Microbiome diversity varies throughout the host gut, increasing from the small to the large intestine. **B***Ascaris* infection is associated with a reduction in microbial diversity at the site of infection in the jejunum. **C** Microbes in the jejunum are major determinants of the composition of the *Ascaris* microbiome. *Ascaris* larvae do not inherit bacteria and thus do not determine the adult worm microbiome. **D** The *Ascaris* microbiome is less diverse than that of its host. Although worm-associated microbes are derived from the host, *Ascaris* has a distinct microbiome. **E** There is no difference in microbial diversity between adult male and female worms, and worm sex was not found to be a major determinant of *Ascaris*-microbiome composition

In conclusion, though host microbes from the immediate surroundings are primary determinants of nematode microbiomes, we provide evidence of bacteria that characterizes either the local microbiome at the site of infection of the host or the inner microbiome of the *Ascaris* worms inhabiting it (summarized as a graphical overview in Fig. 6). An enrichment of specific ASVs in the *Ascaris* gut suggests that the *Ascaris* intestine is a unique niche which may support the growth of microbes that are otherwise under-represented in the host gut.

## Discussion

Despite numerous studies showing that intestinal nematode infections lead to alterations in the gut microbiome of the host, the helminths’ microbiomes and their relationship with host microbes are still an understudied aspect of the parasite-host relationship [15, 16]. Previous work has shown that live bacteria can be retrieved and cultured from the intestine of adult ascarids [13, 14]; however, a culture-free assessment of the microbial composition of the *Ascaris* intestine has not yet been reported. Our work characterizes for the first time the microbiome of this parasitic nematode with respect to its porcine host. We assessed diversity differences between hosts and worms. We report that an *Ascaris* infection reduces microbiome diversity at the site of infection, the jejunum, and that the *Ascaris* microbiome is less diverse than its environment. Importantly, we elucidated the parasite gut microbiome and investigated factors which determine its composition. We highlight that bacteria dominant at the site of infection within the individual host are critical determinants of *Ascaris* microbiome composition in contrast to host bacteria in more distant sites. Our data also indicate that worm microbiome composition is independent of worm sex and parasite burden within the host. While we detected shared bacteria between the parasite microbiome and its microbial environment within the host, we also identified bacteria that differentiate bacterial communities of hosts and parasites. Thus, it appears that the parasite intestine is itself a unique environmental niche better suited for the growth of bacterial communities which are otherwise under-represented in the host gut.

In different parasite-host systems, the effects on gut microbiota diversity, intestinal metabolic environment, and even microbiota-induced immunomodulation during helminth infections have been discussed. Previous studies have assessed the impact of *A. suum* on the porcine fecal microbiome. Williams et al. observed increased diversity in the colon at 14 dpi [27], while Wang et al. reported reduced microbial diversity in the *Ascaris*-infected colon at 54 dpi [28]. Our observations concerning host microbiomes are not directly comparable to these two studies as the others studied distal gut regions and feces while we focused directly on the site of infection and compared this to distal gut regions, which were found to be significantly different from the site of infection, the jejunum. We did however observe a trend towards decreased alpha diversity in the infected jejunum. In agreement with Wang et al., reduction in microbiome diversity was not quantitatively correlated with worm burden. This indicates that worms do not actively compete for host bacteria that they take up from the environment. In accordance with Wang et al. who found significant differences between naive and *Ascaris*-infected gut microbiomes [28], we observed a similar trend towards decreased ASV richness in the jejuna of *Ascaris*-infected pigs. Interestingly, Wang et al. reported enrichment of OTUs assigned to *Lactobacillus*, *Megasphaera*, and *Prevotella* [28], while Williams et al. reported a considerable enrichment of *Succinivibrio* [27]. In our study, the infected jejunum was significantly enriched in ASVs assigned to *Lactobacillus*, *Megasphaera*, and *Prevotella* while *Succinivibrio* was found to be one of the top drivers of the observed enterotypes. These findings are consistent with a meta-analysis of human helminth studies which found *Prevotella* and *Succinivibrio* to be strongly associated with *Ascaris* infection [42]. Interestingly, *Prevotella* may be linked to intestinal dysbiosis and mucosal inflammation [43], while *Succinivibrio* are dominant within the intestinal microbiome of Behçet’s syndrome patients with uveitis [44]. Whether the association of *Prevotella* and *Succinivibrio* with *Ascaris* infection has a pathological consequence for the host or simply serves as a microbial signature of an *Ascaris* infection remains to be determined. While all these studies are restricted to genus-level characterization of prokaryotes, these data suggest that when certain genera are present in the host gut, their relative abundances will change in predictable ways. Future studies employing shotgun metagenomic sequencing and metabolomic profiling will build upon these findings by characterizing the functional potential of the microbial communities present within the *Ascaris* and host intestines.

Previous work demonstrated important roles of gut microbes in the hatching of helminth eggs [45], for the establishment [46], and development and fecundity of helminths [10] within the host intestine. Furthermore, we have previously reported diverse antimicrobial and bacterial modulating activities of excreted and secreted products of helminths in vitro, including *A. suum* [10, 29, 47]. Studies in the free-living nematode *Caenorhabditis elegans* demonstrate that in addition to facing infectious challenges in their immediate environment [48, 49], these roundworms have coevolved microbes and also acquire and shape their own intestinal microbiota, a process strongly influenced by their surroundings [50, 51].

The *Ascaris* intestine is indeed a niche for microbes as ex vivo cultured worms treated with antibiotics still retain living bacteria [14]. Through a culture-free approach, we found that the *Ascaris* intestine possesses a less rich bacterial biome than the jejunal environment. Our findings indicate that the main determinants of the *Ascaris* microbiome are the microbial communities residing in the upper intestinal tract of the host of origin, in particular the dominant bacteria in the host’s jejunum. Previous studies suggest worm sex-specific differences in intestinal physiology [52]. Thus, we looked at whether these physiological differences are reflected in the intestinal microbiome of male and female ascarids. Though certain ASVs were differentially enriched between male and female nematodes, akin to the coding RNA findings from Gao and colleagues, male and female worms did not differ in bacterial taxa richness, and worm sex was not a significant driver of *Ascaris* microbiome composition.

Microbial communities within the *Ascaris* intestine are most similar to those found in the duodenum and jejunum of the host of origin, such that the bacterial compositions could be classified into two main enterotypes: those of the small intestine of the host and *Ascaris* and those of the cecum and colon of the respective host. The jejuna of a majority of infected pigs were found to be dominated by *Lactobacillus*, a genus also well represented in the *Ascaris* intestine though we found different variants of *Lactobacillus* between the host and parasite. Furthermore, the *Ascaris* intestine was found to be dominated by six main genera: *Clostridium sensu stricto* 1, *Escherichia-Shigella*, *Lactobacillus*, *Prevotella*, *Romboutsia*, and *Streptococcus*. In partial agreement with a previous study in which bacteria from the *Ascaris* intestine were cultured [13], we also detected *Staphylococcus*, *Streptococcus*, *Escherichia-Shigella*, and *Pseudomonas*. Seventeen ASVs were enriched in the infected jejunum, while 21 unique ASVs were enriched in *Ascaris*. Interestingly, a meta-analysis aimed at defining the core microbiota of the pig gut reported *Prevotella*, *Clostridium*, *Alloprevotella*, and *Lactobacillus*, among others as shared by > 90% of microbiota samples from commercial swine [52]. Thus, the *Ascaris* microbiome is most similar to its host upper intestine but notably distinct from it. As a first report of the *Ascaris* intestinal microbiome, our findings highlight observable differences between the bacteria in the nematode in contrast to those in the host intestine. These data suggest that *Ascaris* recruits its intestinal microbiome from the available microbes in its immediate surroundings. Thus, it seems likely that the helminth intestine itself is a unique environmental niche for specific nematode-microbe relationships, ranging from mutualism to parasitism, as seen for *C. elegans* [23]. The extent to which the nematode regulates the environment of its own intestine and the microbes present therein is an exciting avenue for further research.

Walk and colleagues found that while adult *H. polygyrus* worm-associated microbes were similar to the infected host ileum and dominated by *Lactobacillaceae*, infective larvae-associated microbes were unique and dominated by *Pseudomonadaceae* [53]. The similarity between adult worms’ and host microbiomes is in line with our observations that the *Ascaris* microbiome is closely related to its immediate environment in the jejunum as opposed to more distal gut regions. Whereas *Ascaris* larvae get in contact with host-associated bacteria upon egg hatching inside the host, *H. polygyrus* larvae hatch in the environment where they can acquire microbes independently of the host. In native free-living *C. elegans* worms, the nematode microbiome was found to be highly variable, less diverse than, and largely influenced by, its surroundings as well as by individual bacterial taxa but with a shared small core community between worms [51]. Genetic diversity among worms may also contribute to the individuality of microbiomes in *C. elegans* [50, 51, 54]. In addition, *C. elegans* worms isolated from the wild and enriched for 3 weeks on agar plates with *E. coli* retain similar microbiota to freshly isolated worms [50]. Interestingly, *Ascaris* females can simultaneously mate with multiple males leading to high genetic diversity [55] which may also contribute to microbiome variability between worms. Taken together, these observations raise critical questions about the stability of nematode and helminth microbiomes. Is there a “core” *Ascaris* microbiome, or is it constantly in flux and dependent on the respective life stage and environment? Well-controlled kinetic experiments would be required to determine how the *Ascaris* microbiome changes with the different life stages and their migration. How is the helminth microbiome impacted by dietary changes in the host? Another interesting question is whether the microbiome of the parasite changes with advancing nematode age during this chronic infection where the worm dwells in the host gut for months and years.

Whether helminth microbiomes harbor bacteria that help the worm to grow and survive in its host environment is still not fully understood; nonetheless, previous studies refer to increased immunoregulatory SCFA in the host intestine associated with helminth infections, including those with *A. suum* [11]. While *Ascaris* might produce these metabolites directly ([46], unpublished observations), *Ascaris* infection promotes the outgrowth of SCFA-producing bacteria such as *Lactobacillus*. In the murine small intestine, *Lactobacillus* promotes the establishment of *H. polygyrus* via elevated regulatory T-cell frequencies that might be linked to bacterial immunoregulatory molecules [46]. Our results show that the *Ascaris* microbiome may harbor microbes that participate in host immunomodulation to promote helminth persistence. Thus, helminth infection may support the growth of microbes which promote a less inflammatory gut environment through the production of systemically active metabolites with ramifications for immune pathologies such as allergies and rheumatic diseases. The considerable abundance of various SCFA-producing bacteria such as *Clostridium*, *Lactobacillus*, and *Streptococcus* in the infected host gut and within the nematode presents potential benefits of helminth infection for the host and deserves deeper investigation.

As a niche, there is the potential that the *Ascaris* intestine may retain or carry potential pathogens, for itself and for the host. *Ascaris lumbricoides* obtained from cholera patients was shown to be colonized by *Vibrio cholerae* [57]. Certain genera detected in the nematode intestine, including *Escherichia-Shigella*, *Pseudomonas*, *Staphylococcus*, and *Streptococcus*, tempt us to ask if the *Ascaris* intestine may serve as a niche for potential porcine and human pathogens. Work in *C. elegans* has shown that human-relevant pathogens like *Salmonella* can infect the nematode intestine and serve as a valuable infection model [58]. At present, nothing is known about microbial pathogens of helminths and whether, like *Salmonella*, certain pathogens might infect both the host and the helminth. We can speculate that in cases where commonly co-occurring zoonotic pathogens such as *Campylobacter* and *Salmonella* have also colonized the porcine intestine, the *Ascaris* intestine may provide protection from host immunity and antibiotics. While we have observed key genera present in the *Ascaris* intestine, experimental analysis of the stability of the *Ascaris* microbiota may reveal species and strains that are essential for helminth survival. Ex vivo antibiotic treatment could be used to disrupt the microbiota [59] allowing for studies with experimental microbiomes [50]. Thus, we may discover commensalistic parasite-microbe relationships as well discovering potential microbial pathogens of helminths. Such findings would open the door for novel therapies focused on parasite control via manipulation of the microbiota.

## Conclusions

Our work presents the first characterization of the microbiome of a zoonotic macroparasite in relation to its host. This provides a starting point towards understanding the complex multilateral relationships between helminth parasites, microbes, and their hosts. Our findings suggest that *Ascaris* selectively acquires its own microbiome from the available pool of microbes in its environment within the upper intestinal tract. Furthermore, our data lead us to intriguing new research questions important for further study. An in-depth characterization of the *A. suum* microbiome across different life stages would shed light on the stability of the microbiome of a body-migratory and long-lived parasitic nematode such as *Ascaris*. Future studies should assess the potential of the helminth intestine to serve as a protective niche for different microbes, along with determining which microbes are beneficial and harmful to the worm. The characterization of helminth microbiomes is a crucial step towards disentangling the mechanisms driving microbiome variation in infected hosts. Understanding parasite-microbiome interactions may aid in predicting disease outcomes and designing novel parasite control strategies.

## Supplementary Information

**Additional file 1: Figure S1. ***Ascaris* larvae lack an inherited microbiome. **Figure S2.** The extent of alterations in richness at the site of infection is not dependent on worm burden. **Figure S3.** Enterotype classification based on the Dirichlet multinomial mixture model. **Figure S4.** Bacterial composition in different gastrointestinal compartments from infected and non-infected pigs.

**Additional file 2: Table S1.** Individual animals, parasite burden, samples per region, and *Ascaris* intestines included in the microbiome analysis. **Table S2.** Core ASVs by sample type. **Table S3.** Permutational analysis of variance for bacterial taxa composition in different gastrointestinal compartments from *Ascaris* infected pigs. **Table S4.** Permutational analysis of variance for bacterial taxa composition in jejunum and *Ascaris* from infected pigs. **Table S5.** Permutational analysis of variance for bacterial taxa composition in different gastrointestinal compartments from *Ascaris* infected and non-infected pigs. **Table S6.** GLMM to assess impact of infection status on microbial dissimilarity among host microbiomes. **Table S7.** Significant differentially abundant ASV between male and female worms*.***Table S8.** Significant differentially abundant ASV between Hosts and parasites (*Ascaris*).

## Data Availability

The sequence data supporting the conclusions of this article are available in the SRA database under the BioProject PRJNA822897: *Ascaris*-pig-microbiome. Code for generating figures and the analysis is available at https://github.com/VictorHJD/ascaris-pig-microbiome.

## Abbreviations

ASV: Amplicon sequence variant
BW: Body weight
Chisq: Likelihood ratio chi-squared statistic
Df: Degrees of freedom
DMM: Dirichlet multinomial mixture
DPI: Days post infection
Exp: Experiment
GI: Gastrointestinal
GLMM: Generalized linear mixed models
Inf: Infected
LRT: Likelihood ratio test
MWU: Mann–Whitney *U*
NMDS: Nonmetric multidimensional scaling
Non: Non-infected
PCR: Polymerase chain reaction
PERMANOVA: Permutational analysis of variance
SE: Standard error
SCFA: Short-chain fatty acids
