# Supplementary material for: Guts within guts: the microbiome of the intestinal helminth parasite Ascaris suum is derived but distinct from its host
Source: Microbiome. 2022 Dec 16;10:229. doi: 10.1186/s40168-022-01399-5 (PMC9756626; doi:10.1186/s40168-022-01399-5)
Supplement: Supplementary file 2 — Additional file 1: Figure S1. Ascaris larvae lack an inherited microbiome. Figure S2. The extent of alterations in richness at the site of infection is not dependent on worm burden. Figure S3. Enterotype classification based on the Dirichlet multinomial mixture model. Figure S4. Bacterial composition in different gastrointestinal compartments from infected and non-infected pigs. [file 40168_2022_1399_MOESM1_ESM.docx]

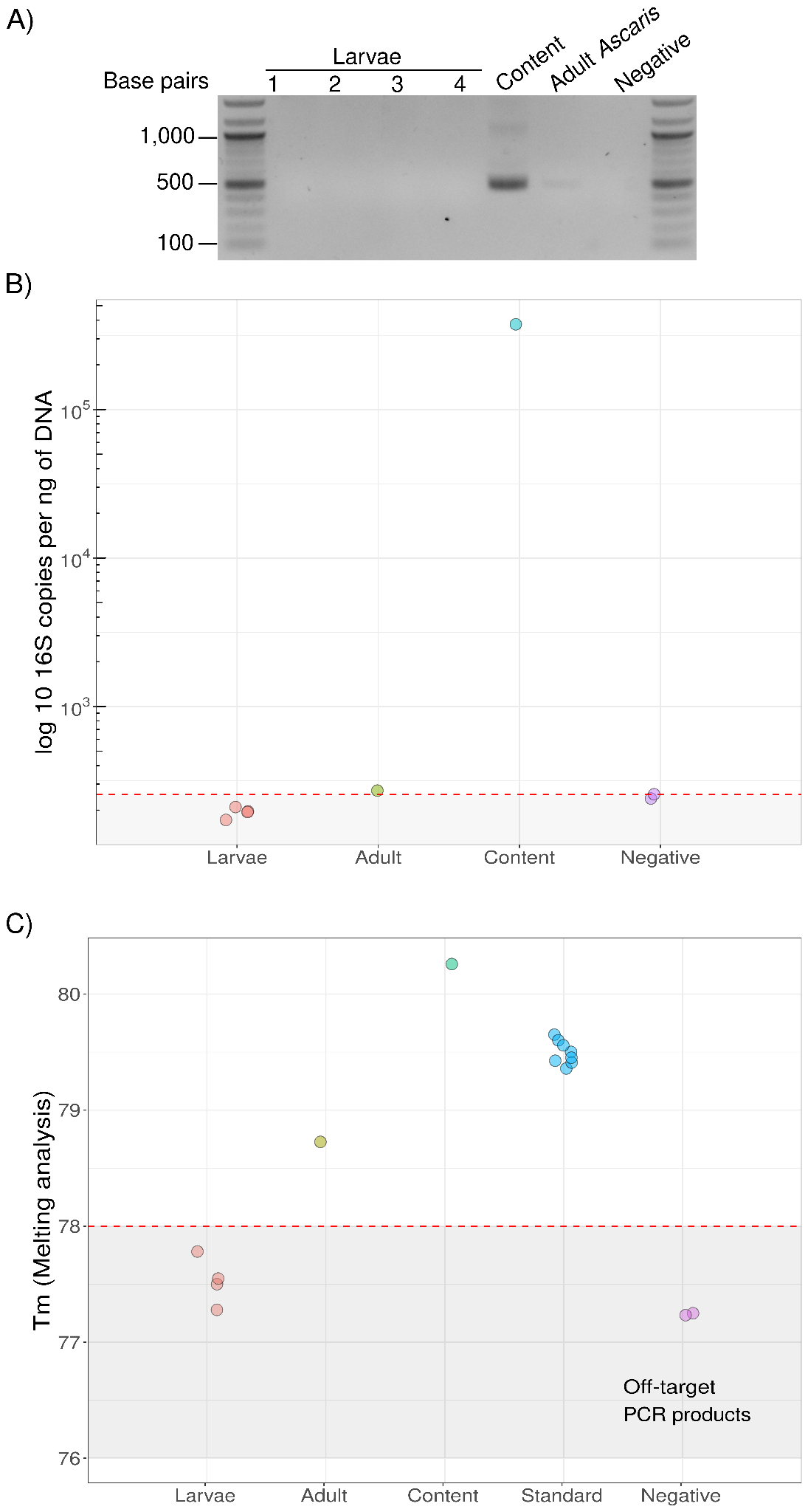


**Supplementary Figure 1. Hatched *Ascaris L3* larvae lack an inherited microbiome. A)** DNA from *in vitro* hatched larvae was also subjected to the two step PCR protocol (16S-specific amplification + barcoding) used for library preparation and loaded together with DNA isolated from jejunal content (Content), DNA isolated from adult *A. suum* (Adult) and non-template control (Negative). Bacterial DNA was not observed in samples from larvae. **B)** Absolute quantification by qPCR did not detect 16S bacterial DNA from *in vitro* hatched larvae. All quantifications were comparable to off-target noise also detected in the negative controls and far below the levels observed for an *Ascaris* adult or jejunum content. **C)** Melting curve analysis confirmed that residual amplification products in larvae are non-specific and also observed in the negative controls. The latter shows the absence of bacterial DNA in *Ascaris* larvae.

**
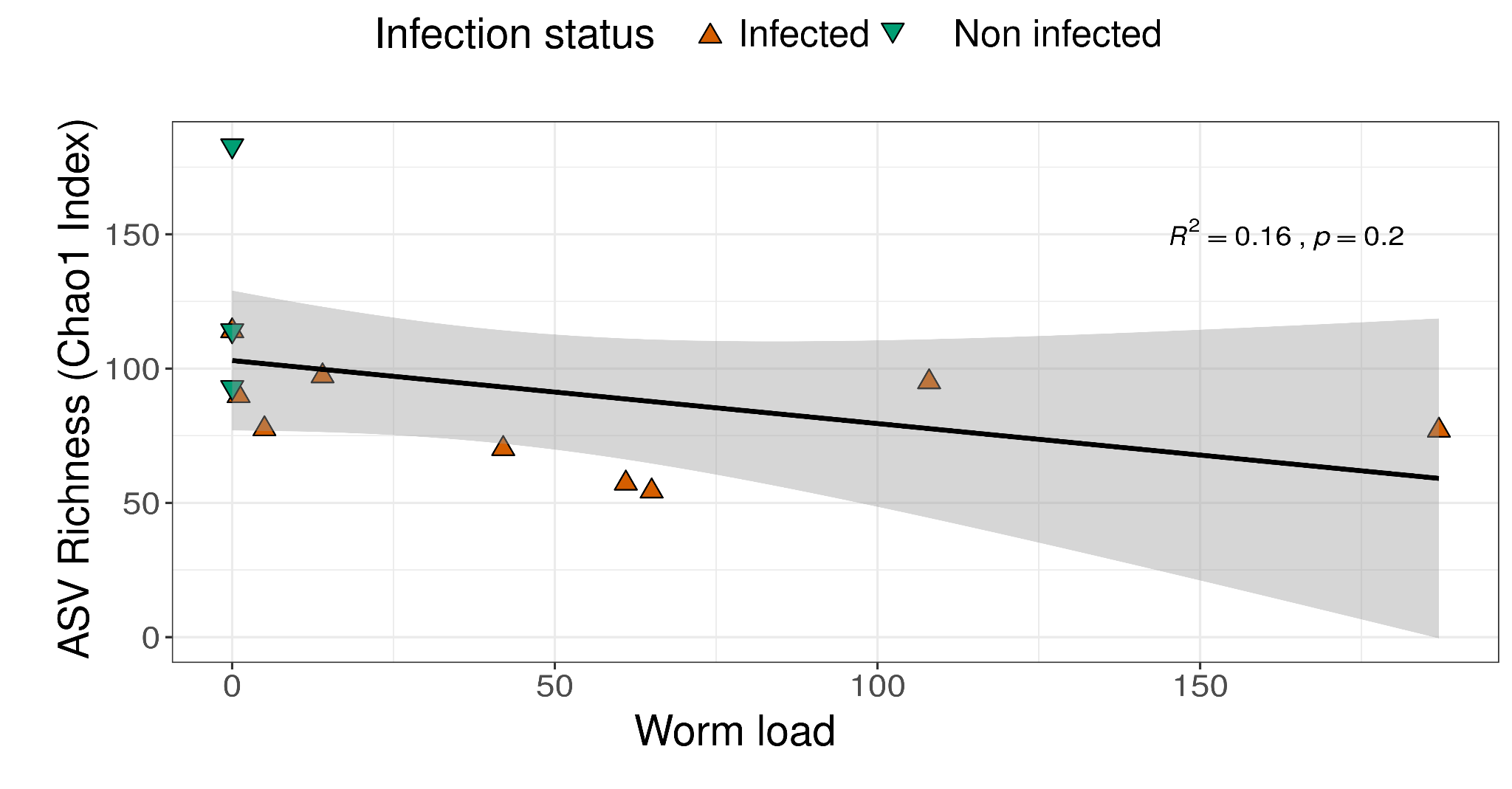
**

**Figure S2. The extent of alterations in richness at the site of infection is not dependent on worm burden.** Linear regression was used to predict Chao's richness index (alpha diversity measurement) based on worm load. Worm load did not explain a significant amount of the variance in alpha diversity (*F_Chao_* (1,10)=, *p*= 0.2, *R^2^*= 0.16 , *R^2^_ad_*_j_= 0.08). Though not statistically significant, the regression coefficient indicates that an increase in one unit of worm load represented on average to a decrease in alpha diversity (𝛽_Chao_= -0.235).


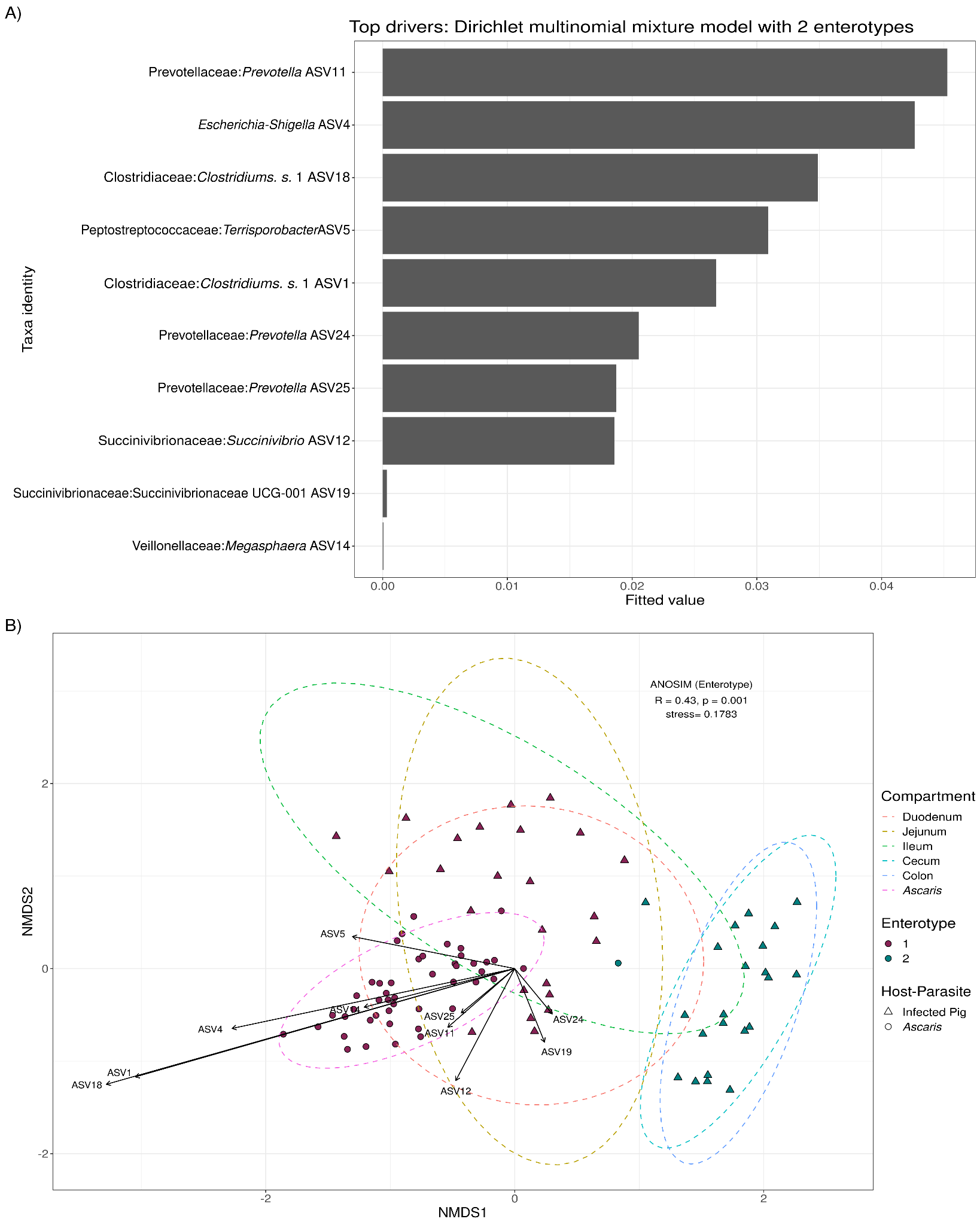


**Figure S3. Enterotype classification based on the Dirichlet multinomial mixture model.** Dirichlet multinomial mixtures (DMM) modeling was applied to the dataset, including infected pigs and their different compartments and *Ascaris* worms. The entire dataset formed two distinct clusters based on the lowest Laplace approximation. **A)** Contribution of each taxonomic group (ASV) to the DMM model with two enterotypes. **B)** Nonmetric multidimensional scaling (NMDS) visualization of DMM clusters using Bray–Curtis distance of gut bacteria. Duodenum, jejunum and ileum (upper GI tract), and cecum and colon (lower GI tract). Each triangle in the graph represents an individual host, while circles represent individual worms, and distances between points are proportional to their biological dissimilarity. Color of the points indicates the enterotype and the dotted lines surrounding them represent the clusters by compartment.The ANOSIM statistic R closer to 1 with <0.05 *p*-value suggests significant separation of microbial community structures into one enterotype for upper gastrointestinal tract and *Ascaris*, and a second enterotype for lower gastrointestinal tract. The stress value being lower than 0.2 indicates a good representation in reduced dimensions.


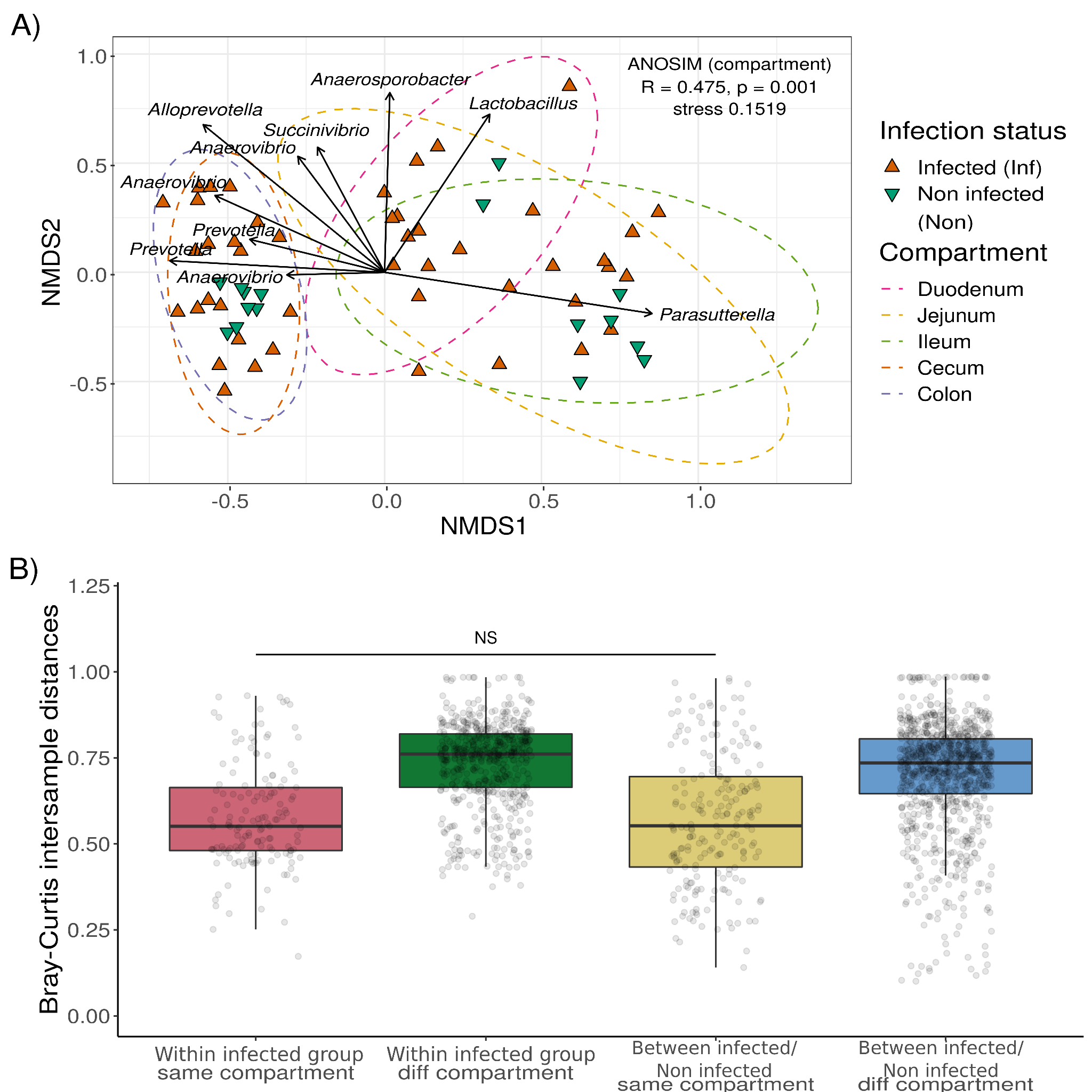


**Figure S4. Bacterial composition in different gastrointestinal compartments from infected and non-infected pigs A)** Nonmetric multidimensional scaling (NDMS) showing differences in microbial composition among gastrointestinal compartments: duodenum, jejunum and ileum (upper GI tract), and cecum and colon (lower GI tract). Each triangle in the graph represents an individual, and distances between triangles are proportional to their biological dissimilarity, calculated with the Bray-Curtis index. Color and shape of the triangle indicates the infection status and the dotted lines surrounding them represent the clusters by compartment. **B)** Pairwise comparison of intersample Bray-Curtis distances within the same compartment shows no difference between infected (Inf) and non-infected (Non) pigs. Every dot represents the distance between a pair of samples. **C5:** Bray-Curtis dissimilarity between infected and non-infected pigs from the same or different compartments. Bray-Curtis dissimilarity within infected or non-infected, from the same or different compartments.
