# Supplementary material for: Guts within guts: the microbiome of the intestinal helminth parasite Ascaris suum is derived but distinct from its host
Source: Microbiome. 2022 Dec 16;10:229. doi: 10.1186/s40168-022-01399-5 (PMC9756626; doi:10.1186/s40168-022-01399-5)
Supplement: Supplementary file 3 — Additional file 2: Table S1. Individual animals, parasite burden, samples per region, and Ascaris intestines included in the microbiome analysis. Table S2. Core ASVs by sample type. Table S3. Permutational analysis of variance for bacterial taxa composition in different gastrointestinal compartments from Ascaris infected pigs. Table S4. Permutational analysis of variance for bacterial taxa composition in jejunum and Ascaris from infected pigs. Table S5. Permutational analysis of variance for bacterial taxa composition in different gastrointestinal compartments from Ascaris infected and non-infected pigs. Table S6. GLMM to assess impact of infection status on microbial dissimilarity among host microbiomes. Table S7. Significant differentially abundant ASV between male and female worms.Table S8. Significant differentially abundant ASV between Hosts and parasites (Ascaris). [file 40168_2022_1399_MOESM2_ESM.docx]

**Table S1. Individual animals, parasite burden, samples per region, and *Ascaris* intestines included in the microbiome analysis.**

| **Animal ID** | **Infection** | **Worm burden*** | **Duodenum** | **Jejunum** | **Ileum** | **Cecum** | **Colon** | ***Ascaris* intestines** |
| --- | --- | --- | --- | --- | --- | --- | --- | --- |
| ***Pig 1*** | + | 5 | 2 | 1 | 2 | 4 | 3 | 5 |
| ***Pig 2*** | + | 187 | 2 | 4 | 0 | 4 | 3 | 7 |
| ***Pig 3*** | + | 42 | 3 | 4 | 2 | 4 | 3 | 8 |
| ***Pig 4*** | + | 0 | 2 | 3 | 2 | 3 | 3 | 0 |
| ***Pig 5*** | + | 1 | 3 | 2 | 2 | 3 | 3 | 1 |
| ***Pig 6*** | - | - | 0 | 0 | 2 | 3 | 3 | 0 |
| ***Pig 7*** | - | - | 0 | 4 | 2 | 3 | 3 | 0 |
| ***Pig 8*** | - | - | 1 | 4 | 0 | 3 | 3 | 0 |
| ***Pig 9*** | - | - | 3 | 4 | 2 | 3 | 3 | 0 |
| ***Pig 10*** | + | 65 | 3 | 3 | 3 | 3 | 3 | 6 |
| ***Pig 11*** | + | 61 | 2 | 1 | 3 | 3 | 3 | 6 |
| ***Pig 12*** | + | 108 | 1 | 3 | 3 | 3 | 3 | 6 |
| ***Pig 13*** | + | 14 | 0 | 1 | 0 | 3 | 2 | 3 |
| ***Pig 14*** | + | 28 | 1 | 0 | 3 | 3 | 3 | 5 |

***At the day of dissection. [1]**

**Table S2. Core ASVs by sample type**

| **Tax ID**  **ASV No: Higher taxonomic annotation** | **Jejunum Infected** | **Jejunum Non infected** | ***Ascaris*** |
| --- | --- | --- | --- |
| ASV1:*Clostridium sensu stricto* 1 | X | X | X |
| ASV2:*Lactobacillus* | X | X | X |
| ASV4:*Escherichia-Shigella* | X |  | X |
| ASV5:*Terrisporobacter* | X | X | X |
| ASV6:*Streptococcus* | X | X | X |
| ASV7:*Romboutsia* | X | X | X |
| ASV9:*Turicibacter sanguinis* | X | X | X |
| ASV11:*Prevotella* | X | X | X |
| ASV12:*Succinivibrio* | X | X |  |
| ASV14:Megasphaera | X | X | X |
| ASV17:*Streptococcus alactolyticus* |  | X |  |
| ASV18:*Clostridium sensu stricto* 1 |  |  | X |
| ASV19:Succinivibrionaceae | X | X |  |
| ASV21:*Clostridium sensu stricto* 1 |  |  | X |
| ASV22:*Lactobacillus* | X | X | X |
| ASV24:*Prevotella* | X | X |  |
| ASV25:*Prevotella copri* | X | X | X |
| ASV26:*Anaerovibrio* |  | X |  |
| ASV27:Prevotellaceae | X | X |  |
| ASV28:*Parasutterella* |  | X |  |
| ASV29:*Lactobacillus pontis* | X |  | X |
| ASV30:*Lactobacillus reuteri* | X | X | X |
| ASV31:*Lactobacillus* | X | X |  |
| ASV32:*Prevotella* | X |  |  |
| ASV34:Prevotellaceae | X | X |  |
| ASV35:*Megasphaera* | X | X | X |
| ASV37:*Clostridium s. s.* 1 *butyricum* | X | X |  |
| ASV38:*Anaerovibrio* | X | X |  |
| ASV40:*Lactobacillus* | X |  | X |
| ASV41:*Intestinibacter* | X | X |  |
| ASV42:*Prevotella* | X |  |  |
| ASV44:Prevotellaceae | X |  |  |
| ASV49:Rickettsiales |  |  | X |
| ASV53:*Anaerovibrio* | X |  |  |
| ASV54:*Prevotella* | X | X |  |
| ASV56:*Anaerovibrio* | X | X |  |
| ASV58:*Parasutterella* |  | X |  |
| ASV59:*Prevotella* | X |  |  |
| ASV61:Prevotellaceae | X | X |  |
| ASV62:*Escherichia*-*Shigella* |  |  | X |
| ASV63:*Subdoligranulum* | X | X |  |
| ASV64:Lachnospiraceae | X |  |  |
| ASV71:*Parasutterella* |  | X |  |
| ASV74:Prevotellaceae | X |  |  |
| ASV76:*Prevotella* | X | X |  |
| ASV78:*Prevotella* | X |  |  |
| ASV88:*Blautia* | X | X |  |
| ASV91:*Lactobacillus* | X |  |  |
| ASV131:Clostridia |  | X |  |
| ASV133:*Subdoligranulum* |  | X |  |
| ASV191:*Parasutterella* |  | X |  |
| ASV197:*Bifidobacterium* |  | X |  |

**Table S3. Permutational analysis of variance for bacterial taxa composition in different gastrointestinal compartments from *Ascaris* infected pigs**

|  | **Df** | **Sums of squares** | **Mean Sqs** | ***F*-Model** | **R^2^** | **Pr(>*F*)** |
| --- | --- | --- | --- | --- | --- | --- |
| *Host-Parasite* | 1 | 3.8043 | 3.804 | 26.286 | 0.157 | 0.001*** |
| *Compartment* | 4 | 3.3525 | 0.838 | 5.791 | 0.138 | 0.001*** |
| *Individual* | 9 | 5.8167 | 0.646 | 4.466 | 0.240 | 0.001*** |
| **Residuals** | 78 | 11.2888 | 0.145 | - | 0.465 | - |
| **Total** | 92 | 24.2623 | - | - | 1.000 | - |

---

Significance codes: 0 ‘***’ 0.001 ‘**’ 0.01 ‘*’ 0.05 ‘.’ 0.1 ‘ ’ 1, Df: Degrees of freedom, *F*-Model: *pseudo F*-test statistic, R^2^: Variance explained and *p* value based on 999 permutations.

**Table S4. Permutational analysis of variance for bacterial taxa composition in jejunum and *Ascaris* from infected pigs**

|  | **Df** | **Sums of squares** | **Mean Sqs** | ***F*-Model** | **R^2^** | **Pr(>*F*)** |
| --- | --- | --- | --- | --- | --- | --- |
| *Host-Parasite* | 1 | 1.1339 | 1.133 | 9.212 | 0.093 | 0.001*** |
| *Individual* | 8 | 5.5563 | 0.695 | 5.643 | 0.454 | 0.001*** |
| **Residuals** | 45 | 5.5388 | 0.123 | - | 0.453 | - |
| **Total** | 54 | 16.2289 | - | - | 1.000 | - |

---

Significance codes: 0 ‘***’ 0.001 ‘**’ 0.01 ‘*’ 0.05 ‘.’ 0.1 ‘ ’ 1, Df: Degrees of freedom, *F*-Model: *pseudo F*-test statistic, R^2^: Variance explained and *p* value based on 999 permutations.

**Table S5. Permutational analysis of variance for bacterial taxa composition in different gastrointestinal compartments from *Ascaris* infected and non-infected pigs**

|  | **Df** | **Sums of squares** | **Mean Sqs** | ***F*-Model** | **R^2^** | **Pr(>*F*)** |
| --- | --- | --- | --- | --- | --- | --- |
| *Compartment* | 4 | 4.790 | 1.198 | 9.330 | 0.314 | 0.001*** |
| *Infection Status* | 1 | 1.046 | 1.046 | 8.153 | 0.069 | 0.001*** |
| *Individual* | 12 | 3.749 | 0.312 | 2.434 | 0.246 | 0.001*** |
| **Residuals** | 44 | 5.648 | 0.128 | - | 0.371 | - |
| **Total** | 61 | 15.233 | - | - | 1.000 | - |

---

Significance codes: 0 ‘***’ 0.001 ‘**’ 0.01 ‘*’ 0.05 ‘.’ 0.1 ‘ ’ 1, Df: Degrees of freedom, *F*-Model: *pseudo F*-test statistic, R^2^: Variance explained and *p* value based on 999 permutations.

**Table S6.** **GLMM to assess impact of infection status on microbial dissimilarity among host microbiomes.**

|  | **Estimate** | **SE** | ***t*-value** | **Var explained** | **Chisq** | ***P*-value** |
| --- | --- | --- | --- | --- | --- | --- |
| **Model: Host-Host microbial dissimilarity** | | | | | | |
| Intercept | 0.7258 | 0.0188 | 38.63 | - | - | - |
| ***Same compartment*** | -0.1478 | 0.0087 | -17.084 | 14.7% | 271.14 | **<0.001**** |
| ***Same individual*** | -0.0797 | 0.0157 | -5.068 | 1.7% | 25.25 | **<0.001**** |
| ***Same infection status*** | -0.0342 | 0.0144 | -2.381 | 0.3% | 4.69 | **0.03*** |

---

Significance codes: ‘***’ 0.001, ‘**’ 0.01, ‘*’ 0.05, SE: Standard error, *t*-value: *t*-test statistic, Chisq: Likelihood ratio Chi-squared statistic and *p-*value

**Table S7. Significant differentially abundant ASV between male and female worms**

|  | **log_2_Fold**  **Change** | **Phylum** | **Family** | **Genus/Species** |  |
| --- | --- | --- | --- | --- | --- |
| ASV116 | 27.96 | Bacteroidota | Prevotellaceae | *Alloprevotella* | Ascaris Males |
| ASV79 | 27.24 | Bacteroidota | Prevotellaceae | *Prevotellaceae* NK3B31 group | Ascaris Males |
| ASV78 | 26.74 | Bacteroidota | Prevotellaceae | *Prevotella* | Ascaris Males |
| ASV124 | 26.10 | Firmicutes | Lachnospiraceae | *Roseburia* | Ascaris Males |
| ASV259 | 26.04 | Firmicutes | Clostridiaceae | *Clostridium sensu stricto 1* | Ascaris Males |
| ASV228 | 25.98 | Bacteroidota | Prevotellaceae | *Prevotella* | Ascaris Males |
| ASV73 | 25.85 | Bacteroidota | Prevotellaceae | *Prevotellaceae* NK3B31 group | Ascaris Males |
| ASV210 | 24.90 | Firmicutes | Streptococcaceae | *Streptococcus porcorum* | Ascaris Males |
| ASV108 | 24.85 | Firmicutes | Ruminococcaceae | *Ruminococcus* | Ascaris Males |
| ASV141 | 24.78 | Bacteroidota | Prevotellaceae | *Prevotellaceae* NK3B31 group | Ascaris Males |
| ASV128 | 24.42 | Bacteroidota | Prevotellaceae | *Prevotella* | Ascaris Males |
| ASV133 | 24.41 | Firmicutes | Ruminococcaceae | *Subdoligranulum* | Ascaris Males |
| ASV155 | 24.05 | Firmicutes | Veillonellaceae | *Dialister* | Ascaris Males |
| ASV166 | 23.25 | Bacteroidota | Prevotellaceae | *Prevotellaceae NK3B31 group* | Ascaris Males |
| ASV147 | 21.73 | Fibrobacterota | Fibrobacteraceae | *Fibrobacter* | Ascaris Males |
| ASV156 | -28.84 | Firmicutes | Clostridiaceae | *Clostridium sensu stricto 1* | Ascaris Females |
| ASV51 | -28.43 | Firmicutes | Peptostreptococcaceae | *Terrisporobacter* | Ascaris Females |
| ASV297 | -26.67 | Firmicutes | Clostridiaceae | *Clostridium sensu stricto 1* | Ascaris Females |
| ASV138 | -24.52 | Firmicutes | Selenomonadaceae | *Mitsuokella jalaludinii* | Ascaris Females |
| ASV82 | -24.17 | Bacteroidota | Prevotellaceae | *Prevotella* | Ascaris Females |

**Table S8. Significant differentially abundant ASV between Hosts and parasites (*Ascaris*)**

|  | **log_2_ Fold**  **Change** | **Phylum** | **Family** | **Genus** |  |
| --- | --- | --- | --- | --- | --- |
| ASV203 | 30.00 | Firmicutes | Lactobacillaceae | *Lactobacillus* | Host |
| ASV197 | 29.72 | Actinobacteriota | Bifidobacteriaceae | *Bifidobacterium* | Host |
| ASV119 | 29.71 | Bacteroidota | Prevotellaceae | *Prevotellaceae UCG-001* | Host |
| ASV350 | 29.45 | Actinobacteriota | Atopobiaceae | *Coriobacteriaceae UCG-002* | Host |
| ASV226 | 28.84 | Firmicutes | Peptococcaceae | *Peptococcus* | Host |
| ASV248 | 28.28 | Firmicutes | Veillonellaceae | *Megasphaera* | Host |
| ASV400 | 28.08 | Firmicutes | Lactobacillaceae | *Lactobacillus* | Host |
| ASV455 | 27.34 | Firmicutes | Peptostreptococcaceae | *Asaccharospora* | Host |
| ASV266 | 26.96 | Actinobacteriota | Bifidobacteriaceae | *Bifidobacterium* | Host |
| ASV462 | 26.78 | Firmicutes | Clostridiaceae | *Clostridium sensu stricto 1* | Host |
| ASV430 | 26.49 | Firmicutes | Lactobacillaceae | *Lactobacillus* | Host |
| ASV513 | 26.13 | Firmicutes | Clostridiaceae | *Clostridium sensu stricto 1* | Host |
| ASV1328 | 26.11 | Actinobacteriota | Bifidobacteriaceae | *Pseudoscardovia* | Host |
| ASV426 | 26.00 | Actinobacteriota | Bifidobacteriaceae | *Bifidobacterium* | Host |
| ASV112 | 25.62 | Bacteroidota | Prevotellaceae | *Prevotella* | Host |
| ASV169 | 25.34 | Bacteroidota | Prevotellaceae | *Prevotella* | Host |
| ASV134 | 24.80 | Bacteroidota | Prevotellaceae | *Prevotellaceae NK3B31 group* | Host |
| ASV15 | -30.00 | Firmicutes | Clostridiaceae | *Clostridium sensu stricto 1* | Ascaris |
| ASV156 | -28.60 | Firmicutes | Clostridiaceae | *Clostridium sensu stricto 1* | Ascaris |
| ASV84 | -25.12 | Firmicutes | Lachnospiraceae | *Agathobacter* | Ascaris |
| ASV118 | -24.95 | Bacteroidota | Prevotellaceae | *Prevotellaceae NK3B31 group* | Ascaris |
| ASV141 | -24.63 | Bacteroidota | Prevotellaceae | *Prevotellaceae NK3B31 group* | Ascaris |
| ASV116 | -24.37 | Bacteroidota | Prevotellaceae | *Alloprevotella* | Ascaris |
| ASV124 | -24.16 | Firmicutes | Lachnospiraceae | *Roseburia* | Ascaris |
| ASV73 | -23.73 | Bacteroidota | Prevotellaceae | *Prevotellaceae NK3B31 group* | Ascaris |
| ASV212 | -23.69 | Firmicutes | Lactobacillaceae | *Lactobacillus* | Ascaris |
| ASV66 | -23.30 | Firmicutes | Lachnospiraceae | *Lachnospira* | Ascaris |
| ASV259 | -22.52 | Firmicutes | Clostridiaceae | *Clostridium sensu stricto 1* | Ascaris |
| ASV228 | -22.15 | Bacteroidota | Prevotellaceae | *Prevotella* | Ascaris |
| ASV155 | -21.58 | Firmicutes | Veillonellaceae | *Dialister* | Ascaris |
| ASV367 | -21.48 | Firmicutes | Staphylococcaceae | *Staphylococcus* | Ascaris |
| ASV82 | -21.18 | Bacteroidota | Prevotellaceae | *Prevotella* | Ascaris |
| ASV111 | -21.11 | Bacteroidota | Prevotellaceae | *Prevotellaceae NK3B31 group* | Ascaris |
| ASV505 | -21.06 | Firmicutes | Streptococcaceae | *Streptococcus* | Ascaris |
| ASV215 | -20.40 | Firmicutes | Ruminococcaceae | *Ruminococcus* | Ascaris |
| ASV315 | -19.77 | Firmicutes | Lachnospiraceae | *Anaerosporobacter* | Ascaris |
| ASV171 | -11.77 | Proteobacteria | Pseudomonadaceae | *Pseudomonas* | Ascaris |
| ASV4 | -2.65 | Proteobacteria | Enterobacteriaceae | *Escherichia-Shigella* | Ascaris |
